# Developmental cell fate choice in neural tube progenitors employs two distinct *cis*-regulatory strategies

**DOI:** 10.1016/j.devcel.2022.11.016

**Published:** 2022-12-13

**Authors:** M. Joaquina Delás, Christos M. Kalaitzis, Tamara Fawzi, Madeleine Demuth, Isabel Zhang, Hannah T. Stuart, Elena Costantini, Kenzo Ivanovitch, Elly M. Tanaka, James Briscoe

**Affiliations:** 1 The Francis Crick Institute, 1 Midland Road, London NW1 1AT, UK; 2 Institute of Molecular Pathology (IMP), Vienna Biocenter (VBC), 1030 Vienna, Austria

## Abstract

In many developing tissues, the patterns of gene expression that assign cell fate are organized by graded secreted signals. *Cis*-regulatory elements (CREs) interpret these signals to control gene expression, but how this is accomplished remains poorly understood. In the neural tube, a gradient of the morphogen sonic hedgehog (Shh) patterns neural progenitors. We identify two distinct ways in which CREs translate graded Shh into differential gene expression in mouse neural progenitors. In most progenitors, a common set of CREs control gene activity by integrating cell-type-specific inputs. By contrast, the most ventral progenitors use a unique set of CREs, established by the pioneer factor FOXA2. This parallels the role of FOXA2 in endoderm, where FOXA2 binds some of the same sites. Together, the data identify distinct *cis*-regulatory strategies for the interpretation of morphogen signaling and raise the possibility of an evolutionarily conserved role for FOXA2 across tissues.

## Graphical abstract

**Graphic abstract:**
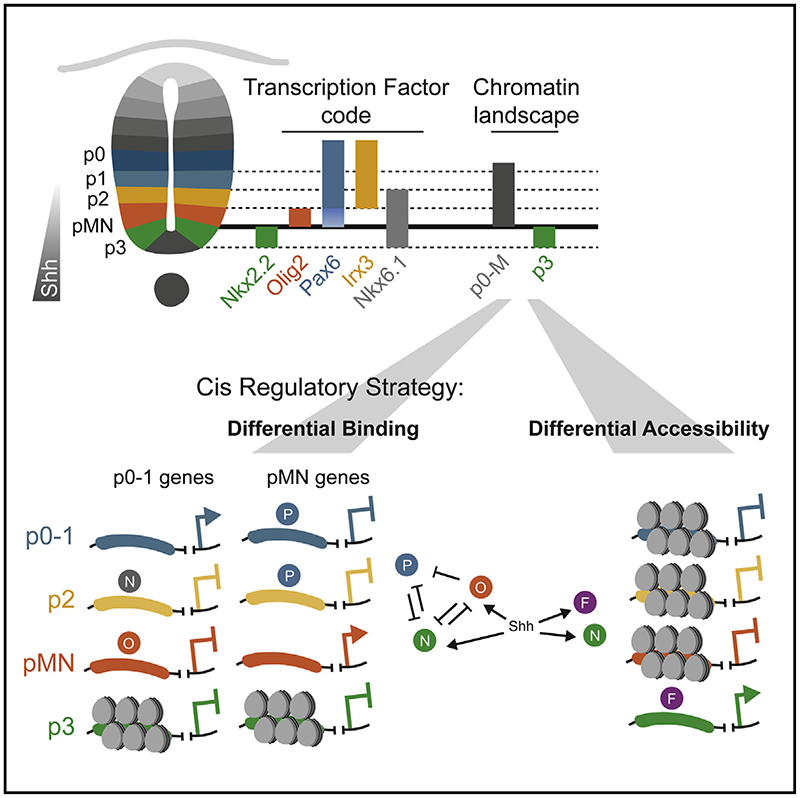

## Introduction

During development signaling cues direct cell fate decisions. Precise spatiotemporal gene expression is essential for this process. *Cis*-regulatory elements (CREs) integrate inputs from tissue and cell-type-specific transcription factors (TFs), as well as signaling effectors, to direct gene expression.^1^ This suggests a straightforward mechanism in which distinct signals produce different cell types by activating different transcriptional effectors that bind to different CREs. However, in many tissues, a single signal directs multiple, alternative cell fates.^2^ Two strategies can be envisioned for how CREs interpret a single signal to define multiple cell fates. Different CREs could function in different cell types. In this strategy—differential accessibility—the availability of an element is the principal determinant of cell identity. In an alternative strategy—differential binding—the same CREs could be used in all cell types in the tissue and the configuration of proteins bound to the elements determines cell fate. An implication of these two strategies is that choosing between two fates will require chromatin remodeling in the case of the differential availability of CREs but can happen without remodeling in the case of different protein configurations at the CREs.

**Figure 1:**
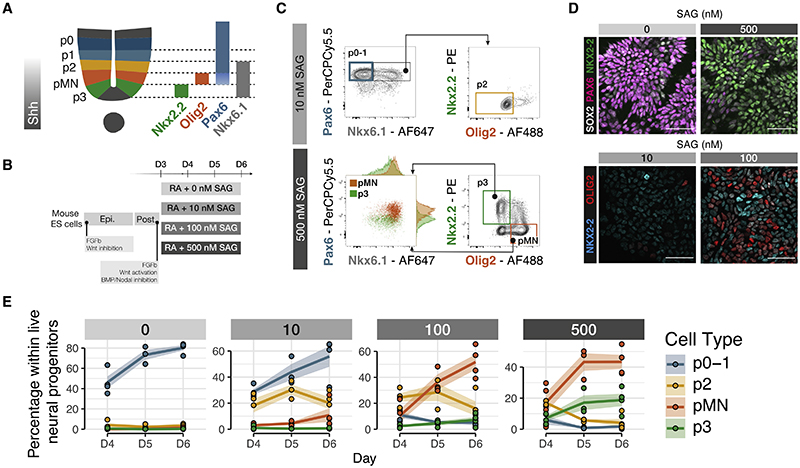
A stem cell model of ventral neural tube progenitors (A) Schematic of ventral spinal cord progenitors and the markers used for the combinatorial multi-color flow cytometry and sorting strategies. (B) Schematic of the protocol for the differentiation of mouse ES cells to generate ventral neural progenitors following equivalent signaling cues to embryonic development. (C) Representative flow cytometry plots of the gating strategies used for both analysis and sorting of NPs p0-1, p2, pMN, and p3. Cells were gated as NPs by excluding dead cells and selecting SOX2+. (D) Representative immunohistochemistry of ES cells differentiated for 6 days show expression PAX6 when exposed to 0 nM SAG and NKX2.2 if exposed to 500 nM. At 100 nM SAG, both OLIG2 or NKX2.2 are detected compared with little or no signal at 10 nM SAG. Scale bars, 50 μm. (E) Proportion of NPs at each SAG concentration shows generation of higher proportions of more ventral cell types at increasing SAG concentrations. Dots are individual samples. n = 4 biological replicates for each SAG concentration. Line represents the average. Shaded areas, SEM. SAG, smoothened agonist. See also Figure S1.

A well-characterized example of a signal controlling multiple cell types is the morphogen sonic hedgehog (Shh). Shh controls the pattern of neuronal subtype generation in ventral regions of the developing neural tube.^3^ Shh, initially secreted from the notochord and later from the floor plate (FP),^4,5^ forms a ventral to dorsal gradient in the neural tube and establishes a set of molecularly distinct domains that occupy characteristic positions along the dorsal-ventral axis. Progenitors in each domain differentiate into distinct classes of post-mitotic neurons. This organization is necessary for the subsequent assembly of the locomotor and sensory circuits.^6,7^ The progenitor domains are identifiable by characteristic gene expression programs (Figure 1A). NKX2.2 is expressed in p3 progenitors, the most ventral domain;^8^ OLIG2 is expressed in adjacent motor neuron progenitors (pMNs);^9^ PAX6 is expressed in more dorsal progenitor domains, including p0, p1, and p2 domains, whereas lower expression is detected in pMN;^10^ NKX6.1 is expressed in, p3, pM, and p2 domains.^11^

The pattern of progenitor gene expression is determined by a gene regulatory network (GRN). Broadly expressed tissue-wide activators, such as SOX2, promote the transcriptional programs of multiple progenitor domains.^12–14^ Concurrently, a set of TFs encoding Groucho/TLE-dependent transcriptional repressors are differentially expressed in distinct progenitor domains.^15^ Pairs of these TFs, expressed in adjacent domains, cross-repress each other and form a network of transcriptional repression that selects a single progenitor identity by repressing all other cell fates.^16–18^ The transcriptional effectors of Shh signaling, the GLI (Gli1, Gli2, and Gli3) proteins provide the spatially polarized input that initiates the patterning process.^19–21^ The combination of the positive and negative inputs generated by this network establish and position the discrete boundaries of gene expression domains in response to graded Shh signaling.^11,22,23^

Although the genetic architecture of the GRN is well established, how cell fate choice is implemented through CREs is less clear. Enhancer elements that drive expression of domain-specific TFs have been identified.^13,14^ The presence of these cell-type-specific enhancer elements could support the differential accessibility strategy in which different CREs are available in different cell types. However, many of these CREs are bound by SOX2, the Shh effector GLI1 and the repressive TFs expressed in alternative cell types, including NKX2.2, OLIG2, and NKX6.1.^13,16,17^ This suggests that the same CREs are employed in different cell types and the composition of TFs at these CREs determines activity: the differential binding strategy.

To distinguish between these two regulatory control strategies, we made use of neural progenitors (NPs) differentiated from embryonic stem (ES) cells.^24,25^ We reasoned chromatin accessibility was the best approach to capture the global landscape of functional regulatory regions. We therefore needed a cell-type-specific accessibility assay, in a system without cell surface markers. Here, we developed an ATAC-seq work-flow—crosslinked and TF-sorted ATAC-seq (CaTS-ATAC) — based on intracellular flow sorting that allowed the assay of chromatin accessibility of NP cell types. We found that most NPs shared a common set of CREs. These elements are bound by the known repressive TFs, indicating that these cell fate decisions are made by changing the composition of proteins bound to the elements. By contrast, the most ventral NP cell type, p3, has a distinct regulatory program. We identify the role of FOXA2 in driving this chromatin remodeling and show that it is required for p3 specification. This is reminiscent of the pioneering role of FOXA2 in endoderm lineages, raising the intriguing possibility that it represents a remnant of a GRN co-opted from this germlayer or a shared evolutionary origin for these cell types.

## Results

### A stem cell model of ventral neural tube progenitors

To generate NPs of different dorsoventral identities, we made use of an established protocol for the directed differentiation of mouse ES cells.^24,25^ To mimic graded Shh signaling, we used different concentrations, ranging from 0 to 500 nM of the Shh agonist SAG (smoothened agonist) (Figures 1A and 1B). We observed a dose response as measured by Shh target genes *Gli1* and *Ptch1* (Figure S1A). Using markers for specific progenitor domains,^26^ we assayed cell identity with single-cell resolution (Figures 1C and 1D). Each SAG condition generated a mixture of two or three NP types. Using the combinatorial expression of TFs, we quantified the proportions of different cell types for each concentration by intracellular staining followed by flow cytometry (Figure 1C). This showed enrichment of the expected cell types. The highest concentration (500 nM SAG) produced the highest percentage of the most ventral domain, p3, characterized by the expression of NKX2.2; pMN, which express the TF OLIG2, were produced at 500 and 100 nM SAG; p2 progenitors, which express NKX6.1 and PAX6, were produced at 100 and 10 nM. PAX6 expression in the absence of NKX6.1 identifies p0 and p1 progenitors, and these were produced at 0 and 10 nM SAG. The expected combinatorial expression of TFs was also observed: All p3 and pMN cells also expressed NKX6.1 (Figure 1E). Moreover, we observed a lower level of PAX6 in pMN cells compared with p3 cells, consistent with *in vivo* data (Figure S1B).

The different SAG conditions also generated the expected neuronal subtypes after the addition of Notch inhibitor dibenzazepine (DBZ), which induces neuronal differentiation (Figure S1C). Overall, the data indicate that the ES-derived model of neural tube patterning ventral of progenitors faithfully mimics the *in vivo* response of NPs to Shh.

### CaTS-ATAC identifies cell-type-specific regulatory program

**Figure 2:**
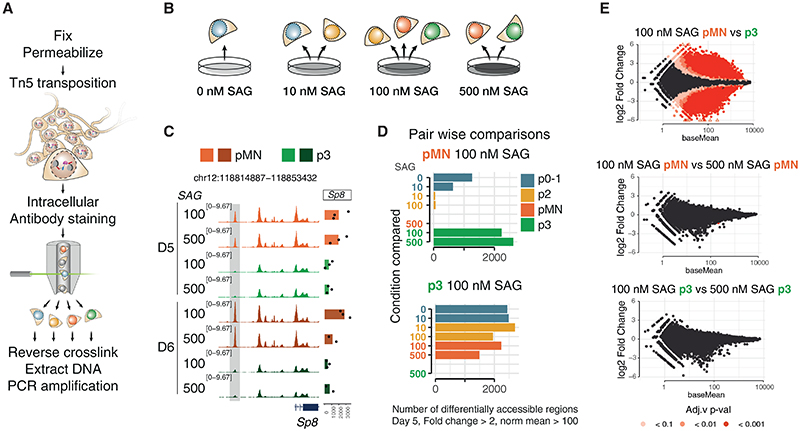
Chromatin accessibility reflects cell-type identity independent of SAG concentration (A) Schematic of CaTS-ATAC, a strategy for cell-type-specific ATAC-seq based on intracellular markers developed for this study. (B) Cell types analyzed from each SAG concentration. (C) Representative genome coverage plot of a differentially accessible region and expression of the nearby gene, *Sp8*, shows accessibility is consistent for each cell type regardless of the SAG concentration from which it was generated. (D) Quantification of differentially accessible regions between the indicated sample and all other samples at day 5 of differentiation shows no significant differences with the same cell type generated from a different SAG concentration. Thresholds used: absolute fold change > 2, basemean > 100. (E) MA-plot (log_2_-fold change versus base mean) for the indicated comparisons show large numbers of differentially accessible elements between different cell types generated under the same SAG concentration, but not between the same cell type generated in different SAG concentrations. n = 3 biological replicates. See also Figure S2.

To distinguish whether the gradient of Shh signaling is interpreted via differential accessibility or differential binding, we reasoned we needed global chromatin accessibility information with cell-type specificity. ATAC-seq is conventionally performed in live, permeabilized cells. However, the cell-type-specific markers that distinguish NPs are intracellular and therefore cannot be used on live cells. Based on previous work,^27^ we devised a strategy—CaTS-ATAC—to perform ATAC-seq on formaldehyde fixed cells. This involved intracellularly staining and flow cytometry sorting of cells followed by bulk ATAC library preparation (Figure 2A; STAR Methods).

We assessed the overall quality of the method by comparing it to ATAC-seq from live cells using the Omni-ATAC protocol.^28^ To this end, we sorted Olig2-expressing cells using a previously developed reporter cell line^25^ and compared it with CaTS-ATAC pMN samples, both from 100nM SAG at day 5 of differentiation. CaTS-ATAC data were of high quality as assessed by the insert size distribution, proportion of mitochondria DNA, and FRiP (Fraction of Reads in Peaks) score (Figures S2A–S2C). There was some underrepresentation of the smallest size fragments, which we assume is due to the reverse crosslinking steps, as noted by others^29^ (Figure S2A). The correlation between samples from the same method was high (R^2^ > 0.94) (Figures S2D and S2E) and a bit lower between methods (R^2^ > 0.72 for any pair wise comparison) (Figure S2F). We note that the samples will be slightly different as the reporter-sorted cells will include newly differentiated MNs and some OLIG2+ NKX2.2+ double positive cells, which are excluded from out CaTS-ATAC pMN. Overall, we were satisfied with the quality assessments and used standard ATAC QC metrics for all further samples.

We performed CaTS-ATAC-seq over a 4-day time course following the addition of different concentrations of SAG. For each condition, we sorted the two or three predominant cell types. This allows us to compare the regulatory landscape of the same cell type originating at different SAG concentrations as well as different cell types within the same dish (Figure 2B).

To explore this dataset, we first examined the correlation between samples based on accessibility at transcriptional start sites (TSSs) or distal elements (not overlapping with TSS). As previously reported in other systems,^30^ accessibility of distal elements exhibited greater cell-type specificity and higher dynamic range than promoter elements (Figures S2G and S2H). This confirms our previous observation that ATAC-seq can capture the regulatory landscape of NPs,^31^ and it can do so with cell type specificity.

### Cell-type-specific accessibility configuration is independent of Shh agonist concentration

We first investigated the effect of SAG concentration on cell type-specific chromatin accessibility. To do this, we compared global accessibility changes between different NP types generated under the same SAG concentration and the same NP type generated under different SAG concentrations.

We found that the accessibility profiles between the same NP type isolated from different SAG concentrations were indistinguishable (Figures 2C–2E). Differential accessibility analysis using DESeq2 revealed no significant differentially accessible peaks between pMN derived from different SAG concentrations (Figures 2D and 2E) nor between p3 derived from different SAG concentrations (Figures 2D and 2E). By contrast, over 2,000 differentially accessible peaks were identified between pMN and p3 generated under the same SAG concentration (thresholds: fold change over 2, FDR < 0.01 and base mean > 100 normalized counts) (Figure 2D). Similarly, p0-1 or p2 arising from different concentrations had few if any differentially accessible elements (Figure S2I, arrowhead), whereas almost 500 elements were highly differentially accessible between these two NP types generated from the same SAG concentration (Figure S2I).

Thus, each cell type, identified by marker gene expression, acquired a specific chromatin landscape, irrespective of the agonist concentration to which they were exposed. This indicates the GRN mechanism of morphogen interpretation, that converts graded Shh input into distinct cell identities, establishes not only the discrete transcriptional identities of NPs but also their chromatin landscapes. Thus the differential chromatin accessibility induced by different levels of Shh signaling reflect cell-type-specific identity and are a product of the GRN that patterns progenitors.

### A shared chromatin landscape for p0-1, p2, and pMN

**Figure 3:**
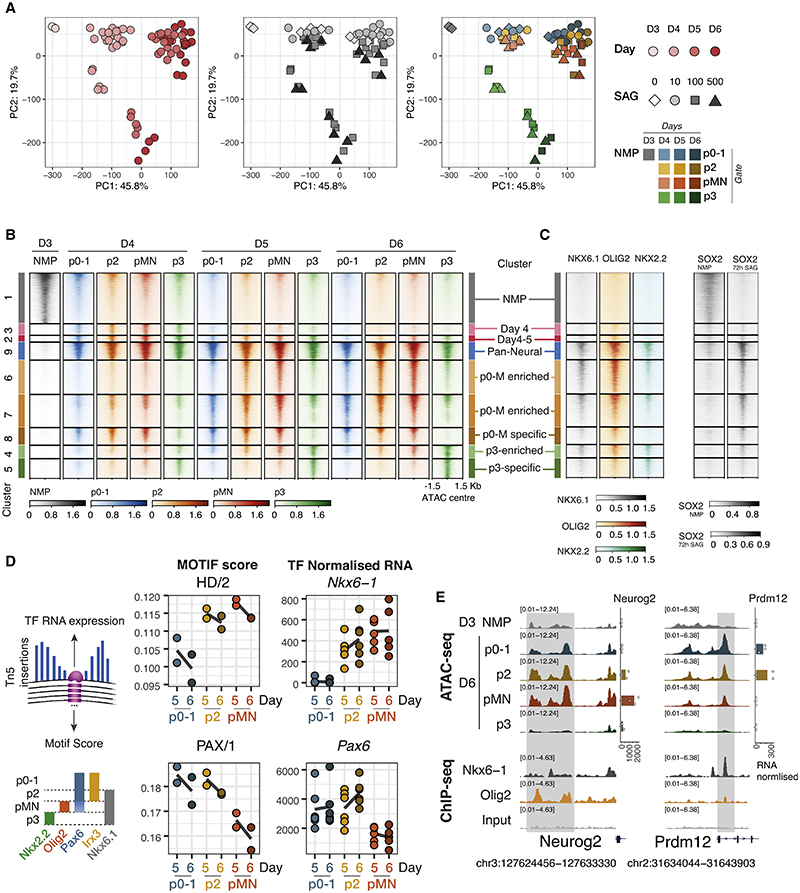
Two regulatory landscapes underlie the Shh response of neural progenitors (A) Dimensionality reduction (principal component analysis) based on the most variable 30,000 consensus elements shows two different behaviors: a shared one for p0-1, p2, pMN samples, and a different one for p3 samples, regardless of SAG concentration. Each symbol represents a sample colored by differentiation day, SAG concentration, or cell type as indicated in the legend. n = 3 biological replicates (independent differentiations). (B) Heatmap showing ATAC-seq coverage for elements differentially accessible between any two cell types or time points with the same dynamics, grouped in clusters (see STAR Methods) shows decommissioning of the NMP program (cluster 1), a shared pan-neuronal cluster (cluster 4) and two behaviors in cell type-specific accessibility, a shared for p0-1, p2, and pMN samples (clusters 6–8), and a unique program for p3 (clusters 4 and 5). Elements ordered by average accessibility. (C) Heatmap of ChIP-seq coverage for the same elements for NKX6.1, OLIG2, and NKX2.2^16^ performed in neural embryoid bodies treated with SAG shows binding to both pan-neuronal and cell-type-specific elements, and SOX2 from either NMP stage^32^ or SAG-treated neural embryoid bodies^13^ correlates with accessibility in either NMP or neural progenitors, respectively. (D) Footprinting scores (using TOBIAS, see STAR Methods) for motifs with highly variable scores between p0-1, p2, and pMN at days 5 and 6, and normalized RNA counts for the most correlated TF within the motif archetype. The motifs for cell-type-specific TFs show expected footprinting differences. (E) Example loci that are accessible across p0-pMN but were not accessible at day 3 and do not became accessible in p3 NPs. ChIP-seq for NKX6.1 and OLIG2^16^ shown for the same elements. Normalized expression for nearby genes *Neurog2* and *Prdm12* shown. Points are individual samples; bars represent average expression. See also Figure S3.

To examine the global accessibility dynamics, we performed principal component analysis (top 30,000 elements, Figure 3A) and clustered differentially expressed elements across all conditions using k-means (Figure 3B; STAR Methods). The main change across all data is the global decommissioning of the neuromesodermal progenitor (NMP) program and establishment of the NP program, which occurs between day 3 and day 4 of differentiation (Figures 3A and 3B). This involves closing a large proportion of elements open in D3 NMPs (Figure 3B, cluster 1 “NMP”), as well as opening elements in NPs (Figure 3B, cluster 3 “day 4,” cluster 2 “days 4 and 5,” cluster 9 “pan-neural”). This is expected as cells adopt neural identity. Consistent with this, SOX2 ChIP-seq from NMPs^32^ is enriched in cluster 1 NMP elements, and SOX2 ChIP-seq from neural cells^13^ is enriched in neural clusters (9, 6, 7, 8, 4, and 5) (Figure 3C).

From day 4 onward, as cells adopt one of the ventral NP fates, the analysis revealed that pMN, p2 and p0-1 were remarkably similar (Figures 3A and 3B). This is surprising as these progenitors are molecularly distinct and give rise to functional different neurons: pMNs, characterized by the expression of OLIG2 generate motor neurons, whereas the p2 domain expresses IRX3 and generates V2 interneurons. RNA expression of marker genes from paired RNA-seq samples confirmed the expected identity of these cell types (Figure S3A). NP p3 were, however, distinct in their chromatin accessibility profile (Figures 3A and 3B).

The markers that define NP domains are repressive TFs and are directly involved in cell-type specification. OLIG2 is expressed in the pMN and represses p3 and p2 fate. NKX6.1 is expressed in p2, pMN and p3 and represses p0-p1. Binding data (ChIP-seq) from these TFs^16^ showed both NKX6.1 and OLIG2 bind regions of open chromatin accessible across all the panneural (cluster 9) and regions shared by pMN, p2, and p0-1 (p0-M enriched/specific clusters 6, 7, 8) (Figures 3C, S3B, and S3C). Since these TFs are only expressed in a subset of the cell types, this suggests that the same elements are open across multiple cell types and occupied by different TFs in different NP subtypes.

To explore gene regulation differences between p0-p1, p2, and pMN NPs, we turned to footprinting. This computational approach uses transposase insertion sites to identify motifs that are under-transposed within open chromatin and are thus likely to be protected by a bound protein. We used TOBIAS^33^ with motifs from three databases,^34–36^ grouping motifs into published archetypes based on position weight matrix clustering of motifs.^37^ The analysis revealed homeodomain (HD) and PAX motifs among those most differentially scoring between pMN, p2, and p0-1 at day 5 and day 6 (Figure 3D). From all the TFs associated with these motifs, the genes *Nkx6-1* and *Pax6* had RNA expression levels most consistent with the footprint score for HD and PAX, respectively (Figure 3D). In addition, we found the Ebox motif to be among the most variable footprints between these NPs (Figure S3D). The high footprint score in pMN is expected as these NP express *Olig2*. However, this is not the only Ebox binding TF expressed in these cell types that could explain this footprint. *Neurog2* and *Neurod1* both bind the same motif and are expressed in p2 and pMN, potentially explaining the Ebox footprint score in p2 NPs. This highlights the challenges in assigning footprints to specific TFs. Overall, the differential footprints found between p0-1, p2, and pMN supports the differential binding strategy, in which a common set of open chromatin regions is shared across cell types and different protein composition of the CREs determine gene activity (Figure 3E).

### FOXA2 establishes the p3 regulatory landscape

By contrast to the other NPs, p3 cells had a distinct global accessibility profile, consistent with the differential accessibility strategy (Figures 3A and 3B). Accessibility was apparent at a unique set of open chromatin regions not shared with any other NP (“p3-specific” cluster 5), and at a set of elements highly enriched in this cell type (“p3-enriched” cluster 4). These differences are evident in the second principal component of the PCA where p3 cells are clearly distinct from all other ventral progenitors (Figure 3A).

Analysis of ChIP-seq for the p3-specific TF NKX2.2 revealed NKX2.2 was enriched in the p3-enriched (cluster 4) and p3-specific clusters (cluster 5) (Figures 3C and S3B). However, NKX2.2 was not specific, and it also bound the elements shared across all cell types (pan-neural cluster 9) and, to a lesser extent, the p0-pMN-enriched clusters (clusters 6 and 7) (Figures 3C, S3B, and S3C). Thus, the expression of NKX2.2 did not appear to explain the distinct chromatin landscape of p3. What then drives this distinct chromatin remodeling?

**Figure 4:**
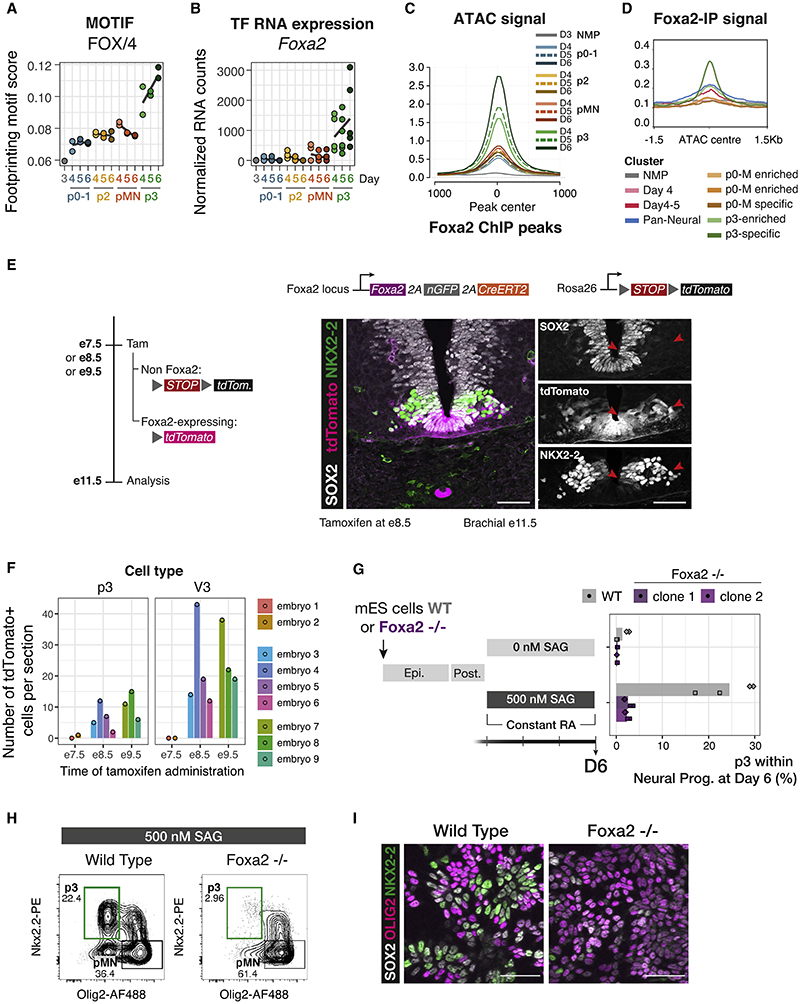
FOXA2 drives the p3-specific chromatin accessibility program (A) Footprinting score for the FOX motif is highest in p3 samples. (B) *Foxa2* expression in p3 NPs suggests it is the most likely candidate to drive the footprinting signal. (C) Average ATAC-seq accessibility at FOXA2 ChIP-seq peaks^13^ in the indicated samples shows these regions are highly accessible in p3 NPs. (D) Normalized FOXA2 ChIP-seq coverage showing accessibility in p3-specific elements from the groups of ATAC-seq elements in the indicated clusters from Figure 3B. (E) Genetic lineage tracing indicates that cells that expressed *Foxa2* at E8.5 (tamoxifen administration) have contributed to the p3 progenitor and V3 neuronal cell types by E11.5 (red arrows). (F) Quantifications of p3 and V3 cells expressing tdTomato in embryos collected after tamoxifen administration at the indicated times. Biological replicates: n = 2 for E7.5, n = 4 for E8.5, n = 3 for E9.5. (G) *Foxa2*−/− ES cells fail to generate p3 NPs when exposed to 500 nM SAG. (H) Representative flow cytometry plots of the quantifications in (G) showing a marked reduction in p3 NPs from *Foxa2*−/− ES cells compared with wild type. Cells are gated for SOX2+ live neural progenitors. (I) Representative immunohistochemistry staining for SOX2, OLIG2 and NKX2,2 showing reduced number of cells expressing NKX2.2 in *Foxa2*−/− mutant cells at day 6 of differentiation treated with 500 nM SAG. Scale bars, 50 μm. See also Figure S4.

To explore which TFs made p3 NP different from other NPs, we compared footprinting scores across all conditions. The strongest footprint distinguishing p3 from other NPs was predicted to correspond to FOXA2 (Figures 4A, 4B, and S4C). This was intriguing as FOXA2 is known to be expressed during FP development.^38–40^ FOXA2 responds to Shh,^41^ and it induces Shh expression,^42^ thus generating a new signaling center in the ventral neural tube.

To exclude the possibility that the cells isolated as p3 NPs (SOX2+ NKX2.2+ OLIG2–) contained FP cells, we devised culture conditions that promoted expression of mature FP markers (*Arx*, *Shh*) in which *Foxa2* is expressed at high levels. We compared our spinal cord (SC) differentiation with FP differentiation and observed little expression of FP markers in SC conditions even at the last time point sorted, D6 (Figure S4A). Moreover, the expression of FP markers even at later time points was very inefficient in SC conditions, suggesting that the FOXA2 footprint is not the result of FP induction (Figures S4A and S4B).

We next analyzed published FOXA2 ChIP-seq generated from neuralized embryoid bodies treated with SAG.^13^ This revealed FOXA2 binding only to p3-specific peaks (Figure 4D). Consequently, we observed open chromatin in p3 NP samples in the regions bound by FOXA2 (Figure 4C). This contrasts with ChIP-seq of other NP TFs, which showed accessibility across all NP cell types (Figure S3C). Together, the data prompt the hypothesis that FOXA2 is driving the unique chromatin accessibility profile we observe in p3 cells.

### *In vivo* p3 cells have a history of FOXA2 expression

If FOXA2 plays a role in generating the p3 chromatin accessibility profile, it should be expressed in p3 cells. *In vivo* NKX2.2 and FOXA2 are co-expressed at early stages of neural tube development,^43,44^ but these FOXA2 expressing cells have been thought to mark the future FP.^45^ To determine whether cells that express FOXA2 also contribute to the p3 domain, we took advantage of genetic lineage tracing in mouse embryos. Foxa2-driven CreERT2 expression^46^ induced recombination of a fluorescent reporter when tamoxifen was administered to pregnant mice at E8.5 or E9.5. Embryos analyzed at E11.5 showed expression of the reporter in both p3 (as identified by SOX2 and NKX2.2 expression) and V3 neurons (identified as laterally located cells expressing NKX2.2 alone) (Figures 4E and 4F). When tamoxifen was administered at E7.5, zero and one p3 cells were detected in two embryos, consistent with *de novo Foxa2* expression induced by ventral Shh signaling (Figure 4F). This demonstrates that p3 cells and their neuronal progeny express *Foxa2* during their history and supports the hypothesis that FOXA2 expression establishes a unique chromatin landscape in p3.

### FOXA2 is required for p3 cell fate specification

Our results are consistent with the hypothesis that FOXA2 drives extensive remodeling of the chromatin landscape in p3 cells, which in turn is essential for p3 identity. To test this hypothesis, we used genome engineering to create a FOXA2 knockout mouse ES cell line. *Foxa2*−/− cells showed a marked reduction in the proportion of p3 cells (25% in wild type to 2% in the mutants) under 500 nM SAG (Figures 4G–4I).

Cells co-expressing NKX2.2 and OLIG2 were still present in *Foxa2*−/− (Figure 4H). This indicates FOXA2 is not directly required for the expression of NKX2.2. Instead, we hypothesize that FOXA2-driven remodeling is required in order for NKX2.2 to exert its repressive activity on OLIG2 and establish the p3 domain. Consistent with this, we observed binding of FOXA2 at the same locations as NKX2.2 (Figure S4D).

### FOXA2 expression substitutes for Shh signaling early in p3 specification

FOXA2 is expressed in NPs closest to the source of Shh, and it is only co-expressed with NKX2.2 at early stages of neural tube development. To ask whether early exposure to Shh signaling is necessary to establish a p3 identity, we took advantage of our *in vitro* NP differentiation protocol to investigate the timing requirements for FOXA2 expression and Shh signaling in p3 specification. To this end, we exposed cells to 0 nM SAG for 24 h before changing regime to 500 nM SAG. Compared with a constant exposure to 500 nM SAG, cells exposed to delayed SAG are greatly impaired in their generation of p3 NPs (Figures S5A and S5B). This was not due to reduced Shh transduction (Figure S5B). The reduction in p3 specification was reminiscent of the outcome of exposing *Foxa2*−/− NPs to 500 nM SAG (Figures 4G and 4H) as we also saw NPs co-expressing NKX2.2 and OLIG2 (Figure S5B). When cells were exposed to SAG from day 3, a proportion of them show low but detectable levels of FOXA2 after 24 h. In the delayed SAG addition conditions, however, cells did not upregulate FOXA2 in the equivalent time frame (Figure S5D).

**Figure 5:**
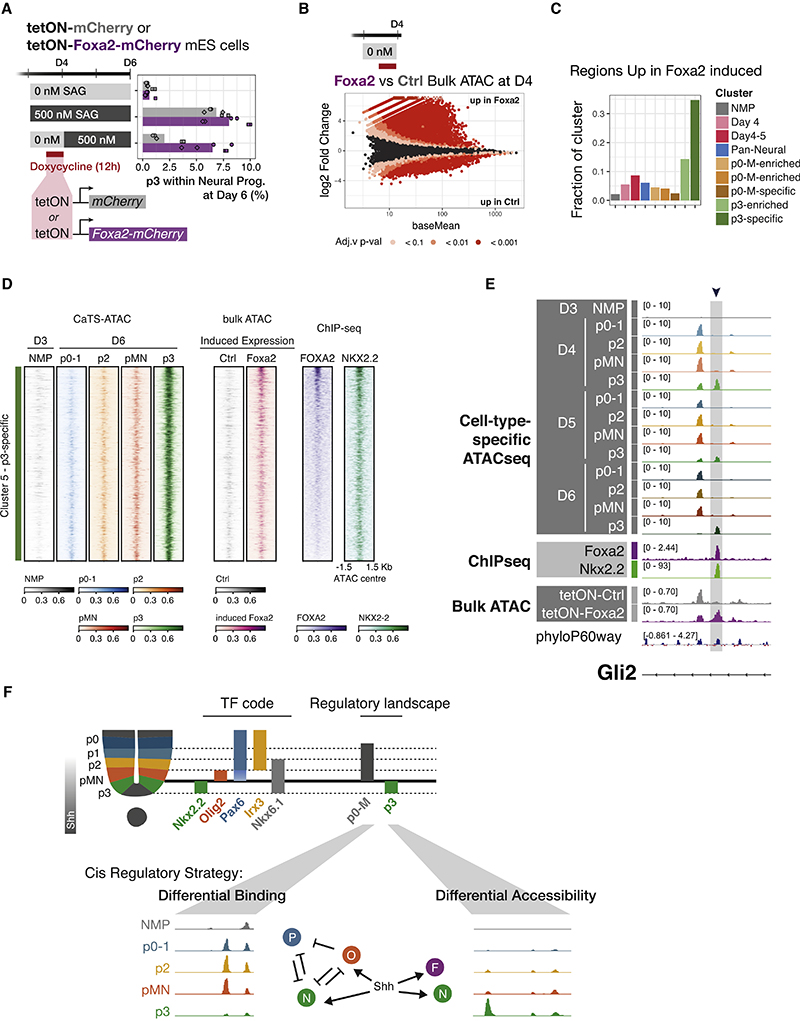
FOXA2 can replace Shh in early p3 specification where it opens p3-specific regulatory elements (A) A delayed SAG regime greatly reduces p3 generation in control cells (mCherry). A 12 h overexpression of tetON-Foxa2-mCherry rescues p3 generation. n = 3 biological replicates (independent differentiations), and 2 independent samples from each differentiation, for each condition. (B) Differential accessibility in Foxa2 forced expression versus control mCherry shows FOXA2 predominantly opens elements. n = 3 biological replicates (independent differentiations). (C) Overlap of differentially upregulated regions from (B) with ATAC clusters (Figure 3B) reveals a large fraction of the p3-specific cluster is opened upon Foxa2 overexpression. (D) Heatmap showing CaTS-ATAC-seq coverage over all sites in the p3-specific cluster 5, bulk ATAC-seq coverage after induced expression of control or FOXA2, as well as ChIP-seq coverage for FOXA2 and NKX2.2. Regions are ordered by FOXA2 ChIP-signal and depict higher accessibility when FOXA2 (but not control) is induced, concomitant with increased binding of FOXA2 and NKX2.2 in the same regions. (E) Example element in a *Gli2* intron with p3-specific chromatin accessibility, FOXA2 and NKX2.2 binding that gains accessibility upon *Foxa2* overexpression but not control. (F) Diagram describing the two regulatory landscapes that underlie the molecular identity of ventral neural progenitors, a differential binding strategy is used to distinguish p0-1, p2, pMN, whereas p3 employ a differential accessibility strategy. See also Figure S5.

This regime provided the opportunity to test whether FOXA2 expression within the initial 24 h of NP differentiation was sufficient to rescue p3 generation. We used doxycycline inducible lines that expressed either mCherry or a Foxa2-mCherry fusion under the control of the doxycycline-responsive promoter tetON. Consistent with prior experiments, overexpression of mCherry alone in this “delayed SAG” regime showed a reduction in p3 generation at D6 (Figure 5A). By contrast, enforced expression of *Foxa2* for 12 h during the initial 24 h of neural differentiation rescued p3 generation to levels comparable with those observed with constant 500 nM SAG (Figure 5A).

These data indicate that high Shh at the onset of SC progenitor specification is required to induce *Foxa2.* This expression of FOXA2 is sufficient to confer competence for Shh signaling to specify p3 identity.

### FOXA2 is sufficient to remodel the chromatin landscape

To test whether FOXA2 is responsible for the early p3-specific chromatin remodeling, we took advantage of the tetON cell lines. We performed bulk ATAC-seq at D4, which is 24 h after the induction of neural identity and 12 h after doxycycline exposure (Figure 5B). Both control and FOXA2-overexpressing conditions showed accessibility across the previously defined NMP and pan-neuronal clusters (Figure S5F). Although neither cell line had been exposed to SAG, FOXA2-expressing cells displayed opened chromatin across the p3-specific cluster (Figure S5F).

Consistent with its known role as a pioneer factor,^47^ FOXA2 overexpression resulted in chromatin opening (Figure 5B). Over 30% of the regions in the p3-specific cluster overlapped with regions more accessible in FOXA2-overexpressing cells (Figure 5C). Increased accessibility in cluster 5 (p3-specific) regions in response to induced FOXA2 is observed together with binding of p3-specific TF NKX2-2, with areas of highest binding also sharing FOXA2 binding (Figure 5D). An example of this behavior is shown for a *Gli2* intron (Figure 5E), as well as three additional loci (Figure S5G). These results support a model in which FOXA2 opens chromatin regions required for the p3 fate. Thus, Shh induction of *Foxa2* at early neural developmental stages is required to configure the p3-specific response of the cells to Shh. Cell fate choice between p0-1, p2, and pMN employ a differential binding strategy, whereas p3 rely on differential accessibility mediated by pioneer factor FOXA2 (Figure 5F).

### A common lineage-pioneering role for FOXA2 across germ layers

FOXA TFs are required for the development of endoderm-derived lineages, pancreas, liver, and lung, where they play partially redundant roles.^48–53^ Although many TFs play roles in multiple tissues, they are generally thought to act via different enhancers in a combinatorial fashion with other tissue-specific TFs.^54,55^ Pioneer TFs are proposed to act by opening up compacted chromatin, thus allowing other TFs to bind.^56^ Hence, a given pioneer TF could have access to the same binding sites independent of the cellular context. However, the binding of pioneer TFs has been shown to depend on epigenetic features.^57^ Indeed, cell-type-specific co-factors direct cell-type-specific binding of FOXA2 in endogenously expressing cell lines and in FOXA2 overexpressing cell lines.^58^

**Figure 6:**
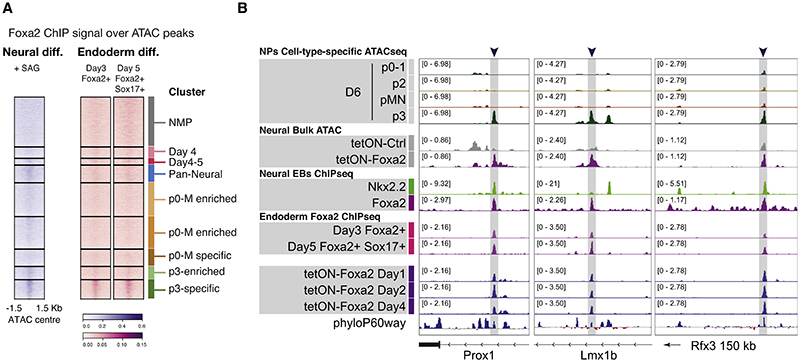
A common regulatory role of FOXA2 in ventral neural and endoderm lineages (A) Heatmap of ChIP-seq coverage for neural FOXA2 and endoderm FOXA2 at two differentiation time points^50^ show binding for both in the p3-specific accessibility cluster. (B) Potentially functionally relevant target genes expressed and/or required in both tissues, with p3-specific accessibility, opened in response to FOXA2 overexpression, bound by FOXA2 in neural and endoderm, including overexpression of FOXA2. See also Figure S6.

We therefore asked how much of FOXA2’s pioneering activity is tissue specific between endoderm and neural p3 progenitors. We examined the binding of FOXA2 in cellular differentiation models of endoderm at day 3 and day 5^50^ and asked whether endoderm FOXA2 was bound to any of the SC differentiation clusters. To our surprise, we found FOXA2 also bound to the p3-specific cluster of elements in endoderm (cluster 5) (Figure 6A). This included genes such as *Prox1*, expressed in both lineages;^59,60^ *Lmx1b*, required for specification of hindbrain p3-derived serotonergic neurons^61,62^ and for *in vitro* pancreatic islet generation;^63^ and *Rfx3*, also required for pancreatic endocrine differentiation^64^ and expressed in the ventral neural tube^65^ (Figure 6B). This argues for a functional role in both tissues for at least some of these targets. Moreover, many of these sites were either opened in NPs upon brief overexpression of *Foxa2* (Figures 6B and S5F) or occupied by overexpressed FOXA2 in endoderm differentiation (Figure 6B).

Although endoderm and NPs also have tissue-specific FOXA2 binding, these data raise the possibility that the two tissues share a core set of targets. Mechanistically, this could be mediated by the classical pioneer role of FOXA2 in this subset of targets or by the presence of common co-factors in both tissues. Intriguingly, it could offer evidence of an evolutionarily conserved GRN.

## Discussion

Here, we identify two *cis*-regulatory strategies that direct cell fate choice in developing NPs. One strategy—differential binding — relies on a common regulatory landscape. The different composition of TFs at these CREs dictates differential gene expression and cell fate decisions. The second strategy—differential accessibility—relies on cell-type-specific chromatin remodeling (Figure 5F). This is the case for p3 NPs, which have a unique set of accessible elements that distinguish them from all other NPs. We show that FOXA2, expressed early in response to Shh, is responsible for the remodeling that opens p3-specific elements. Many of these elements then bind the p3-specific TF, NKX2.2. Intriguingly, a subset of the elements bound and opened by FOXA2 in NPs are also bound by FOXA2 during endoderm differentiation, where it plays a parallel role opening elements later required for specific endoderm lineages.^51^ The identification of these two regulatory strategies establishes a framework for classifying the genomic implementation of cell fate-decision mechanisms.

To assay cell-type-specific chromatin accessibility, we combined intracellular flow cytometry with methods developed to measure protein levels at single-cell resolution^27^ that produced high quality chromatin accessibility data for specific cell types.^29^

The method is applicable to other tissues, *in vitro* and *in vivo*, and will be of particular use in cases where fluorescent reporters are not readily available or when the identification of the desired cell types depends on the intersectional expression of multiple markers. Moreover, the approach could be extended to isolate cells based on other intracellular characteristics, such as post-translationally modified signaling effectors.

A common set of pan-neural elements, not accessible prior to neural induction, was apparent in all NPs. These elements were enriched for binding of TFs involved in the neural tube GRN, including SOX2, NKX6.1, NKX2.2, and OLIG2. The existence of this set of elements is reminiscent of the idea of morphogenetic fields—discrete, modular units of embryonic development^66^ that display a coordinated response to patterning signals.^66^ Comparison of chromatin accessibility in the same NP cell type generated using different levels of Shh signaling revealed that cell identity, rather than morphogen concentration, correlated with chromatin accessibility pattern. This indicates that the morphogen interpretation mechanism that converts graded Shh input into distinct cell identities establishes not only the discrete transcriptional identities of NPs but also their chromatin landscapes.

The shared chromatin landscape of the majority of ventral NPs suggests a differential binding strategy governs gene expression selection for these cells. This is consistent with the “selection by exclusion” mechanism that has been proposed previously to explain the specification of progenitor subtype identity by graded Shh signaling.^16,17^ In this view, the positive and negative transcriptional inputs supplied by the GRN are integrated at CREs associated with target genes, which are accessible in NPs, regardless of whether the target gene is active. Thus, cell fate choice does not depend on major chromatin remodeling but instead is determined by the composition of transcriptional effectors bound. These findings challenge the validity of approaches typically used to infer gene regulation and cell identity from genomic data, which equate open chromatin with gene activity and cell fate. Our data demonstrate that a major regulatory strategy—“differential binding”—violates this assumption and indicate the necessity of developing more nuanced methods.

By contrast, the differential accessibility strategy appears to dictate p3 identity. These NPs are distinguished by accessibility at a distinct subset of CREs that depend on FOXA2. Despite the transience of FOXA2 expression in p3 NPs,^67^ accessibility at the CREs opened by FOXA2 persists and are bound by the p3-specific TF NKX2.2. This is similar to the observation that the presence of FOXA is not required to maintain stable epigenetic states in liver cells.^68^ The marked changes in chromatin landscape initiated by FOXA2 thus represents an example of epigenetic memory and demonstrate how the pioneering activity of a TF rewires a GRN to specify a specific cell type.

The different regulatory strategies have important implications for cellular reprogramming. Cell types sharing the same landscape (differential binding) would be relatively plastic, enabling transitions between the different fates by altering the TF configuration on already available CREs. Consequently, the barrier to reprogram cells between such fates would be lower. On the other hand, the differential accessibility strategy involves chromatin remodeling to facilitate the binding of TFs previously unable to bind specific CREs. The expression of the end point TFs alone might not be sufficient for cell fate acquisition if the elements these TFs need to bind are inaccessible. This might explain the inefficiency or incompleteness of some transgene-driven reprogramming approaches that use only the TFs expressed in the final state of the target cell type.^69^ Understanding and recapitulating the epigenetic trajectory cells follow to reach the desired endpoint might improve such differentiation protocols.

Our results are consistent with prior genetic studies showing the p3 NPs are the only NPs that require activating GLI proteins for their specification.^19,70^ The demonstration that FOXA2 expression can substitute for Shh signaling during the early steps of p3 specification indicates that the requirement for activating GLI proteins is for the expression of FOXA2. This requirement restricts p3 induction to the most ventral progenitors in the neural tube. The expression of FOXA2 and NKX2.2 in p3 NPs also appears to contribute to establishing p3 identity by modulating the cell intrinsic response to Shh.^43^ NKX2.2 promotes *Foxa2* via a mechanism involving *Gli3* repression^43^ and FOXA2 binds and opens an intronic region in the gene encoding *Gli2* (Figure 5D). Together, these data suggest that the interpretation of Shh signaling by p3 NPs is distinct from the adjacent p0-pMN region. This distinction involves remodeling the regulatory landscape of p3 NPs.

FOXA2 has been proposed to act as a pioneer factor in the endoderm, where it remodels chromatin to allow the binding of other TFs.^51,56^ Although TFs often have roles in multiple tissues, they are usually thought to act in a combinatorial fashion with other TFs via different elements in different tissues.^54,55,58^ The observation that FOXA2 binds to p3 NP-specific CREs in endodermal cells raises the possibility these two cell types share some aspects of their regulatory control. Consistent with this, pancreas specification, an endoderm derivative, involves many TFs that are also expressed in the ventral neural tube, specifically in the p3 domain.^71^ The potential shared control by FOXA2 of functionally relevant genes in endoderm and neural tissue raises the question of how this might have arisen. The role of FOXA proteins in endoderm development is evolutionarily conserved^72^ and predates the origin of bilaterians. One possibility therefore is the co-option of an endoderm FOXA2 regulatory program into NPs in an ancestor of vertebrates. Alternatively, it could be evidence of convergent evolution or of a common evolutionary origin for endoderm and neural cells. This latter possibility would be supported by recent work suggesting that parts of the digestive system in the cnidaria Nematostella are of ectodermal origin^73^ and that a common progenitor generates neurons and secretory cells in this species.^74,75^ Thus, the shared regulatory features seen in vertebrate endoderm and neural cells might be a vestige of a common evolutionary origin of these cell types. A deeper understanding of the GRNs in neural tissue and endoderm will be needed to explore this possibility. More generally, it will be important to determine whether other tissues use similar cis-regulatory strategies to specify distinct cell types.

## Limitations of the study

In this study, we used *Foxa2* knockout ES cell lines to show a requirement for *Foxa2* in NPs. Whether there is a similar requirement for FOXA2 *in vivo* is not known because the removal of *Foxa2 in vivo* disrupts axis formation and is embryonic lethal at an earlier time point.^76,77^ Additionally, the previously published ChIP-seq data used in this study was generated from a different directed neural differentiation protocol, and there could be differences in binding of the ventral TFs as a result. Finally, although we demonstrate a good correlation between data produced by CaTS-ATAC and conventional ATAC protocols that use live sorted cells, there could be limitations introduced by the paraformaldehyde fixation necessary for CaTS-ATAC. Paraformaldehyde disrupts TF binding to mitotic chromatin, and this might result in a loss of any cell cycle phase specific signal.

## Star★Methods

**Table:** Key Resources Table

Reagent or resource	Source	Identifier
Antibodies		
Sox2-V450	BD Biosciences	Cat#561610; clone O30-678
Pax6-PerCPCy5.5	BD Biosciences	Cat#562388; clone 018-1330
Nkx6.1-AlexaFluor647	BD Biosciences	Cat#563338; clone R11-560
Goat anti-Olig2	R&D	Cat#AF2418
Nkx2.2-PE	BD Biosciences	Cat#564730; clone 74.5A5
Tubb3-Biotin	BD Biosciences	Cat#560394; TUJ1
Foxa2-AlexaFluor488	SC Biotechnology	Cat#sc-374376 AF488; clone H-4
Goat anti-Sox2	R&D	Cat#AF2018
Rat anti-Sox2	Invitrogen	Cat#14-9811-80, clone Btjce
Mouse anti-Nkx2.2	BD Biosciences	Cat#564731, clone 74.5A5
Rabbit anti-Olig2	Merck Millipore	Cat#AB9610
Rabbit anti-Arx	Kind gift from J.Chelly	N/A
Goat anti-Foxa2	R&D	Cat#AF2400
Rabbit anti-Pax6	Merck Millipore	Cat#AB2237
Donkey anti-rat 405	Biotium	Cat#20419
Donkey anti-mouse AF488	Invitrogen	Cat#A21202
Donkey anti-goat AF488	Invitrogen	Cat#A11055
Donkey anti-rabbit AF488	Invitrogen	Cat#A21206
Strep-APC-Cy7	BioLegend	Cat#405208
Donkey anti-mouse AF568	Invitrogen	Cat#A10037
Donkey anti-goat AF568	Invitrogen	Cat#A11057
Donkey anti-mouse AF647	Invitrogen	Cat#A31571
Donkey anti-goat AF647	Invitrogen	Cat#A21447
Bacterial and virus strains		
BL21-Gold(DE3)	Agilent	Cat#230132
Chemicals, peptides, and recombinant proteins		
Recombinant Mouse LIF Protein	Merck	Cat#ESG1107
Dulbecco's Modified Eagle Medium (DMEM) - Knock OUT	Gibco	Cat#10829-018
ES Foetal Bovine Serum (FBS)	Pan Biotech	Cat#P30-2602
Penicillin/Streptomycin	Gibco	Cat#15140122
L-Glutamine	Gibco	Cat#25030024
Non-essential amino acids	Gibco	Cat#11140-035
2-mercaptoethanol	Gibco	Cat#21985-023
0.05% trypsin-EDTA	Gibco	Cat#25300054
Gelatin	Gibco	Cat#G1393-100ML
Advanced DMEM-F12	Gibco	Cat#21331-020
Neurobasal medium	Gibco	Cat#A35829-01
N2	Gibco	Cat#17502001
B27	Gibco	Cat#17504001
Bovine Serum Albumin solution	Merck	Cat#A7979-50ML
Matrigel	Corning	Cat#356231
bFGF	R&D	Cat#100-18B
LGK	Cayman Chemical Company	Cat#1.800.364.9897
CHIR99021	Axon Medchem	Cat#1386
SB-431542	Tocris	Cat#S0400
DMH1	Adooq Bioscience	Cat#A12820
Retinoic Acid (RA)	Sigma	Cat#R2625
Smoothened Agonist (SAG)	Merck	Cat#566660-5mg
DBZ	Biotechne	Cat#4489
Doxycycline	Sigma-Aldrich	Cat#D9891
Tamoxifen	Sigma-Aldrich	Cat#T5648-5G
Lipofectamine 2000	Invitrogen	Cat#11668030
Hygromycin-B	Thermo Fisher	Cat#10687010
Puromycin	Thermo Fisher	Cat#A1113802
LIVE/DEADTM Fixable Dead Cell Stain Near-IR fluorescent reactive dye	ThermoScientific	Cat#L34976
Phosphate Buffer Saline (PBS)	Gibco	Cat#14190-094
Accutase	Gibco	Cat#00-4555-56
Paraformaldehyde (PFA)	ThermoScientific	Cat#28908
Triton-X100	VWR Chemicals	Cat#28817.295
Superscript III reverse transcriptase	Invitrogen	Cat#18080-051
SYBR Green PCR assay	ThermoFisher Scientific	Cat#A25742
ProLong Gold antifade reagent	Invitrogen	Cat#P36930
Igepal CA-630	Sigma	Cat#I8896-100ML
Tween-20	Sigma	Cat#P2287-500ML
Digitonin	Invitrogen	Cat#BN2006
Proteinase K	Ambion	Cat#AM2546
2X PCR Master Mix NEB	NEB	Cat#M0541S
AMPureXP beads	Beckman Coulter	Cat#A63882
Ni-Sepharose column	Cytiva	Cat#17526801
RNasin Plus	Promega	Cat#N2615
Critical commercial assays		
Zymo DNA concentrator kit	Zymo	Cat#D4013
RecoverAll RNA/DNA Isolation Kit	Thermo Fisher Scientific	Cat#AM1975
SMART-Seq HT kit	Takara	Cat#634437
Nextera XT DNA Library Preparation Kit	Illumina	Cat#FC-131-1096
TDE tagmentation enzyme	Illumina	Cat#15027865
2X TD tagmentation buffer	Illumina	Cat#15027866
RNeasy Qiagen kit with DNAse digest	QIAGEN	Cat#74106
Mouse Embryonic Stem Cell Nucleofector™ Kit	Lonza	Cat#VPH-1001
Deposited data		
CaTS-ATAC, live Omni-ATAC, RNA-seq and bulk ATAC-seq	This study	GSE204921
Experimental models: Cell lines		
*Mus musculus* (Male): HM1	Doetschman et al.^78^	N/A
*Mus musculus* (Male): HM1-Foxa2-/-	This study	N/A
*Mus musculus* (Male): HM1-TetON-Foxa2- mCherry	This study	N/A
*Mus musculus* (Male): HM1-TetON- mCherry	This study	N/A
Experimental models: Organisms/strains		
Mouse: Foxa2^nEGFP-CreERT2/^+	Imuta et al.^46^	MGI:5490029
Mouse: R26^tdTomatoAi14/tdTomatoAi14^ (Gt(ROSA)26Sor^tm14(CAG-tdTomato) Hze^	Madisen et al.^79^	MGI:3809524
Oligonucleotides		
qPCR primers used in this study, see Table S1	N/A
Primers for cloning of Foxa2 guides into pX458, see Table S1	This study	N/A
Oligonucleotides for Tn5 assembly, see Table S1	Picelli et al.^80^	N/A
Oligonucleotides for CaTS-ATAC and ATAC-seq library, see Table S1	This study	N/A
Recombinant DNA		
pX458 vector	Ran et al.^81^	Addgene plasmid 48138
PB-tetOn-destination-PGK-hygro	Stuart et al.^82^	N/A
PB-CAG-rtTA3-PGK-puro	Stuart et al.^82^	N/A
Software and algorithms		
nf-core atacseq pipeline	https://nf-co.re/atacseq	https://doi.org/10.5281/zenodo.2634132
DESeq2	Love et al.^83^	https://doi.org/10.18129/B9.bioc.DESeq2
nf-core chipseq pipeline	https://nf-co.re/chipseq	https://doi.org/10.5281/zenodo.3240506
TOBIAS footprinting tools	Bentsen et al.^33^	https://doi.org/10.1038/s41467-020-18035-1
nf-core rnaseq pipeline	https://nf-co.re/rnaseq	https://doi.org/10.5281/zenodo.1400710
R	R Core Team	https://www.R-project.org/
Fiji	Schindelin et al.^84^	https://imagej.net/software/fiji/
Other		
Superfrost Plus slides	Thermo Scientific	Cat#10149870
22-mm-by-50-mm no. 1.5 thickness coverslips	VWR	Cat#631-0138
6-well plates	Corning	Cat#353046
LoBind 1.5 ml Eppendorf tubes	Eppendorf	Cat#Z666548
DNA High Sensitivity DNA Kit	Agilent	Part No 5067-4626

## Resource Availability

### Lead contact

Further information and requests for resources and reagents should be directed to and will be fulfilled by the lead contact, James Briscoe (james.briscoe@crick.ac.uk).

### Materials availability

Murine ES cell lines generated in this study area available upon request.

## Experimental Model And Subject Details

### Lineage tracing of Foxa2-expressing cells

Foxa2-^2a^-^nEGFP-2a-CreERT2/+^;R26^Tomato Ai14/ Tomato Ai14^ embryos were obtained from time matings. Mouse lines Foxa2^nEGFP-CreERT2/+^ (MGI:5490029)^46^ and R26^tdTomatoAi14/tdTomatoAi14^ (Gt(ROSA)26Sor^tm14(CAG-tdTomato) Hze^ (MGI:3809524)^79^ were backcrossed and maintained in a C57BL6 background. Induction of recombination was achieved by tamoxifen administration by oral gavage at 0.08 mg/body weight.

All animal procedures were performed in accordance with the Animal (Scientific Procedures) Act 1986 under the UK Home Office project licenses PP8527846 and PF59163DB. Animals were housed under a 12-h light, 12-h dark cycle at the Francis Crick Institute animal facility. Animals were housed in singly-ventilated cages. The relative humidity was kept at 45 to 65%. Mouse rooms and cages were kept at a temperature range of 20-24°C. Animals had 24 h access to RO water and autoclaved pelleted food. Caging, bedding, nesting materials and enrichments were autoclaved prior to use and cages are changed routinely. A maximum of 5 adult mice were housed per cage if they were over 20 g. Animals were monitored visually daily for health concerns and once a week a full health check was carried out during a discretionary cage change

### Cell lines

WT experiments were performed with the mouse embryonic stem cell line HM1.^78^ All cell lines were maintained at 37°C with 5% carbon dioxide (CO_2_).

### Generation of Foxa2^-/-^ ES cell lines

Generation of Foxa2 knockout cell lines was performed by electroporating two different guides cloned into the pX458 vector^81^ with the AMAXA nucleofector kit (Lonza Cat no. VPH-1001). Cells were sorted for GFP as single cells one day after electroporation. Clonal cell lines were genotyped and two clones were used. Both carried the same deletion.

### Generation of tetON-Foxa2 and control ES cell lines

PiggyBac (PB)-tetOn-destination-PGK-hygro and PB-CAG-rtTA3-PGK-puro vectors were used from Stuart et al.^82^ Gateway cloning (Invitrogen) was used to insert the coding sequence for mCherry or FoxA2:mCherry fusion protein into the tetOn destination vector. Stable transgenic HM1 mouse ES cell lines were generated by transfection as follows: 1 μg PB-tetOn expression vector, 1 μg PB-CAG-rtTA3 vector, 1 μg non-integrating PBase transposase vector and 2 μl Lipofectamine-2000 (Invitrogen) were incubated for 20 min in 100 μl DMEMF12 (Gibco) at room temperature, then applied to 300,000 cells/6well in 1.6 ml medium for 18 h. Selection was applied to transfectants for at least 5 passages prior to use (150 μg/ml hygromycin-B (ThermoFisher) and 1 μg/ml puromycin [ThermoFisher]). Transfection and selection were performed in feeder-free 2iLIF culture conditions as described in Stuart et al.,^82^ then adapted back to feeders and ES cell medium regime as described below for at least 4 passages prior to experiments.

## Method Details

### Cell culture and neural progenitor differentiation

Mouse ES cell lines were maintained in ES cell medium (Dulbecco’s Modified Eagle Medium (DMEM) Knock Out (Gibco, Cat No. 10829-018) supplemented with 10% Foetal Bovine Serum (Pan Biotech, Cat. No. P30-2602), Penicillin/Streptomycin (Gibco, Cat No. 15140122), 2mM L-Glutamine (Gibco, Cat No. 25030024), 2mM Non-essential amino acids (Gibco, Cat No. 11140-035), and 0.1 mM 2-mercaptoethanol (Gibco, Cat. No. 21985-023)) with 1000 U/ml LIF (Chemicon, Int ESG1107) on mitotically inactive primary mouse embryo fibroblasts (feeders). For spinal cord neural differentiation, ES cells were dissociated using 0.05% trypsin-EDTA (Gibco, Cat No. 25300054) and then plated onto 0.1% gelatinised (Gibco Cat no. G1393-100ML) tissue culture plates for three successive periods of 15 mins to remove feeders. Differentiations were carried out in N2B27 media (Advanced DMEM - F12 (Gibco, Cat. No. 21331-020) and Neurobasal medium (Gibco, Cat. No. A35829-01) (1:1), supplemented with 1xN2 (Gibco Cat no. 17502001), 1xB27 (Gibco Cat no. 17504001), 2 mM L-glutamine (Gibco, Cat No. 25030024), 40 μg/ml BSA (Sigma-Aldrich, Cat No. A7979-50ML), and 0.1 mM 2-mercaptoethanol) with the indicated additives. Cells were plated onto 6-well plates (Corning, Cat. No. 353046) precoated in Matrigel (Corning, Cat. No. 356231) diluted 1/50 in Advanced DMEM - F12 at a density of 20,000 cells per 35mm well in 1.5 ml. The media was supplemented on the different days as follows: Day 0 to Day 2, 10ng/ml bFGF (R&D, Cat. No. 100-18B) and 5 μM LGK (Cayman Chemical Company, Cat. No. 1.800.364.9897); Day 2 to Day 3 for 20 h, 10 ng/ml bFGF, 5 μM CHIR99021 (Axon Medchem, Cat. No. 1386), 10 μM SB-431542 (Tocris, Cat. No. S0400), and 2μM DMH1 (Adooq Bioscience, Cat. No. A12820); from Day 3 onwards, 100 nM RA (Sigma, Cat. No. R2625) and either 0 nM, 10 nM, 100 nM or 500 nM SAG (Merck, Cat. No. 566660-5mg). The “delayed SAG” regime consisted of 100 nM RA and 0 nM SAG from Day 3 to Day 4, followed by a constant concentration of RA and increased SAG to 500 nM. Induction of the tetON system was achieved by supplementing the media with 1 μg/ml Doxycycline (Sigma-Aldrich, Cat. No. D9891) for the second 12h of Day 4. Floor plate conditions were achieved by addition of 500 nM SAG on Day 3 without any addition of RA.

### Flow cytometry of intracellular markers

#### Sample collection

1 μl/ml of LIVE/DEADTM Fixable Dead Cell Stain Near-IR fluorescent reactive dye (ThermoScientific, Cat. No. L34976) was added to cells in culture and incubated at 37°C for 30 mins. Cells were washed twice with Phosphate Buffer Saline (PBS) (Gibco, Cat. No. 14190-094), and were dissociated using 0.5ml Accutase (Gibco, Cat. No. 00-4555-56) per well of a 6 well plate incubated 5 min at 37°C. Cell were collected, centrifuged at 400g for 4 min and resuspended in 100μl of 4% paraformaldehyde (PFA) (ThermoScientific, Cat. No. 28908). PFA fixation was carried out for 10 min at room temperature. Cells were washed in PBS and resuspended in 500μl PBS + 0.5% BSA.

#### Staining

1 million cells were stained for flow cytometry analysis in LoBind 1.5 ml Eppendorf tubes. Pellets were resuspended in 0.1ml of PBS + 0.5% BSA and 0.1% Triton-X100 (VWR Chemicals, Cat No. 28817.295) and were incubated on ice for 10 mins. Cells were then incubated with primary antibodies or directly conjugated antibodies diluted in the same buffer for 1.5 h protected from light at room temperature. When necessary, secondary antibodies were incubated under the same conditions for 40 min. Cells were washed in PBS + 0.5% BSA and 0.1% Triton-X100, and resuspended in 300μl of PBS + 0.5% BSA for analysis on a BD Fortessa analyzer (Becton Dickinson). The antibody panels used were as follows: “D-V progenitors”, Sox2-V450 (1:100), Pax6-PerCPCy5.5 (1:100), Nkx6.1-AlexaFluor647 (1:100), Goat Olig2 unconjugated (1:400), Nkx2.2-PE (1:100), and Tubb3-Biotin (1:800) followed by secondaries donkey anti-goat AlexaFluor488 (1:1000) and Strep-APC-Cy7 (1:20,000); “mCherry inducible”, Sox2-V450 (1:100), Pax6-PerCPCy5.5 (1:100), Goat Olig2 unconjugated (1:400), Nkx2.2-AlexaFluor647(1:100), secondaries donkey anti-goat AlexaFluor488 (1:1000); “D-V with Foxa2”, Sox2-V450 (1:100), Pax6-PerCPCy5.5 (1:100), Goat Olig2 unconjugated (1:400), Nkx2.2-PE (1:100) and Foxa2-AlexaFluor488 (1:100), with secondary donkey anti-goat AlexaFluor647 (1:1000). Antibody details in listed in the key resources table.

### RNA extraction, cDNA preparation, and qPCR analysis

Wells were washed with PBS and lysed in 350 μl of RLT buffer (QIAGEN, Cat. No. 1015762) per 35mm well. After cells were mixed, they were collected in a 2 ml RNase-free Eppendorf tube and stored at -20°C. RNA extraction was performed using RNeasy Qiagen kit with DNAse digest (QIAGEN, Cat. No. 74106) as per manufacturer’s protocol. cDNA was synthesized from 1ug of RNA using Superscript III reverse transcriptase (Invitrogen 18080-051) with random hexamers, and qRT-PCR analyses were performed by QuantStudio 12K Flex Real-Time PCR system (ThermoFisher Scientific) using SYBR Green PCR assay (ThermoFisher Scientific, Cat. No. A25742). All experiments were performed in technical duplicates, biological duplicates or triplicates for each time point analysed. Expression values were normalized against β-actin. Primer sequences in Table S1.

### Immunofluorescence staining of cell differentiations

Cells for immunofluorescence staining were cultured in matrigel-coated glass slides in 12-well plates with all the volumes adjusted to achieve the same cell density. Wells were washed with PBS and fixed in 4% PFA at 4°C for exactly 15 mins. Glass slides were transferred to a new 12-well plate for staining. Blocking was performed for 10 mins with PBS + 1% BSA + 0.1% Triton-X100 at room temperature, cells were incubated in PBS + 1% BSA + 0.1% Triton-X100 with primary antibodies overnight at 4°C, with secondary antibodies at room temperature for 1hr, and mounted with ProLong Gold antifade reagent (Invitrogen, Cat. No. P36930). Fluorescent images were taken with Leica SP8 confocal microscope or an Apotome microscope.

The following primary antibodies were used: goat anti-Sox2 (1:500), mouse anti-Nkx2.2 (1:1000), rabbit anti-Olig2 (1:500), rabbit anti-Pax6 (1:1000), rat anti-Sox2 (1:1000), goat anti-Foxa2 (1:500), rabbit anti-Arx (1:1000). Corresponding donkey-raised secondaries were used. Antibody details listed in the key resources table.

### Generation of mouse sections and immunostainings

Mouse spinal cord sections staining were performed as previously described.^85^ Mouse embryos from timed pregnant females were collected and fixed in 4% PFA for 1.5h at 4°C, washed in PBS, and transferred to 15% sucrose in phosphate buffer overnight at 4°C. Embryos were subsequently embedded in gelatin solution (7.5% gelatin, 15% sucrose in phosphate buffer) and snap-frozen in isopentane on dry ice. Transverse cryosections (thickness: 14 mm) were cut using a Leica CM3050S cryostat (Leica Microsystems Limited, Milton Keynes, UK) and placed on Superfrost Plus slides (Thermo Scientific, Cat. No. 10149870). Slides were stored at −80°C until ready to be processed for immunohistochemistry. After immunohistochemistry, 22-mm–by–50-mm no. 1.5 thickness coverslips (VWR, Cat. No. 631-0138) were mounted onto the sections using ProLong Gold antifade reagent. Immunohistochemistry was performed in the same way as for cell culture slides using the same antibodies against Sox2 and Nkx2.2.

### Quantification of lineage tracing from mouse sections

Images acquired on a Leica SP8 40X objective were segmented using the custom scripts described in https://github.com/tiagu/ *Nucleus*. The DAPI signal was used to segment nuclei and signal intensity for all other channels calculated for each nuclei. The raw data and code to reproduce the quantifications of tdTomato p3 or V3 cells can be found in https://github.com/MJDelas/tdTom_LinTracing. In brief, a threshold was found for the intensities of SOX2, NKX2.2 and tdTomato. Cells expressing SOX2 and NKX2.2 were classified as p3. Cells expressing only NKX2.2 were classified as V3. Position was used as a quality control step.

Because of background binding by a mouse raised antibody in mouse sections, NKX2.2 signal emanating from cells far from the p3 domain were excluded from the analysis. The number of either p3 or V3 expressing tdTomato per section were plotted.

### CaTS-ATAC (Crosslinked and TF-Sorted ATAC-seq)

#### Sample collection

Cell type specific ATAC-seq based on intracellular markers was carried out from HM1 differentiations at the timepoints and SAG concentrations indicated. Dishes were washed with PBS and dissociated with 0.5ml Accutase (Gibco, Cat. No. 00-4555-56) per well of a 6 well plate incubated 5 min at 37°C. Cells were collected in Eppendor LoBind tubes (Eppendorf cat. #Z666548), the wells were rinsed with 1ml PBS and the samples were spun at 400 g for 4 min at room temperature. Samples were resuspended in 1μl/ml of LIVE/DEAD™ Fixable Dead Cell Stain Near-IR fluorescent reactive dye as per manufacturer’s instructions. After 30 min incubation on ice protected from light the samples were spun again and resuspended in 300 μl of PBS, followed by addition of 100 μl of PFA to achieve 1% PFA. Fixation was carried out for 15 min with rotation at 4°C. Fixation was quenched with 25 μl of 2M Glycine for 5 min at 4°C with rotation, spun and resuspended in PBS with 0.5% BSA. Cells were counted and 1 million cells were transferred to a new LoBind Eppendorf tube.

#### Transposition

Tn5 transposition was carried out as previously described^28^ with some modifications. Briefly, samples were resuspended in 0.5 ml of RSB buffer (10 mM Tris-HCl pH 7.4, 10 mM NaCl, 3 mM MgCl2) supplemented with 0.1% Igepal CA-630 (Sigma, Cat. No. I8896-100ML), 0.1% Tween-20 (Sigma, Cat. No. P2287-500ML), and 0.01% digitonin (Invitrogen, Cat No. BN2006), and incubated on ice for 3 min. 1 ml of RSB with 0.1% Tween-20 was then added and samples were spun for 10 min at 2000 g and 4°C. The samples were then resuspended in 1 ml of ATAC mix(2X TDE buffer (Illumina), 50 μl TDE (Illumina), 0.01% digitonin, 0.1% Tween-20 and 330 μl of PBS). Transposition was carried out at 37°C with 1000 rpm shaking for exactly 30 min. The reaction was stopped with 30 μl of EDTA, spun at 2000g for 5 min at 4°C and resuspended in 100 μl of PBS with 0.5% BSA and 0.1% Triton X-100.

#### FACS

Flow cytometry staining was carried out as described in “Flow cytometry of intracellular markers”. Samples processed in a BD Fusion cell sorter (Beckton Dickinson). 15,000 cells of each desired population were sorted in to 0.5ml of PBS with 0.5% BSA. Sorted cells were spun for 10 min at 3000 g and 4°C and resuspended in Reverse crosslink buffer (50mM Tris-HCl ph8, 0.5% Tween-20, 0.5% Igepal CA-630) supplemented with 1 μl of 20 mg/ml Proteinase K (Ambion, Cat. No. AM2546) and incubated overnight at 65°C shaking at 300 rpm.

#### DNA isolation

The following day samples were spun down to collect condensation and DNA was isolated using the DNA clean up & Concentrator kit (Zymo, Cat. No. D4013) as per manufacturer’s instructions. DNA was eluted in 21 μl of DNAse-free water. DNA was stored at -20°C until ready to be processed.

#### Library generation

Transposed DNA was first amplified with 2X PCR Master Mix NEB (Cat. No. M0541S), and 1 μl of constant forward primer and indexed reversed primer (Table S1) (both at 25 μM) in a total reaction volume of 50 ul. The program was as follows: 5 min at 72°C, 30 s at 98°C, followed by 7 cycles of 30 s at 98°C, 30 s at 63 °C and 60 s at 72°C, with a final extension of 5 min at 72°C. DNA was cleaned up with 1.8x volumes of AMPureXP beads (Beckman Coulter, Cat. No. A63882) and eluted in 18 μl of EB buffer. qPCR was carried out to determine the number of extra cycles using 2 μl of the amplified DNA in technical duplicates. Each 20 μl reaction contained 20 μl of 2X SYBR Green PCR assay, using PowerUP SYBR Green Master Mix (Thermo Fisher, Cat. No. A25742) and 2.5 μl of each primer (at 25 μM each). The qPCR program was as follows: initial activation for 2 min at 50°C, 30 s at 98°C, followed by 40 cycles of 10 s at 98°C, 30 s at 63°C and 60 s at 72°C. The number of additional cycles was calculated as 1/4 of the maximum amplification.^86^ The second amplification was done using 12.5 μl of amplified DNA, 2X PCR Master Mix NEB and 2.5 μl of each primer (at 25 uM) in a total volume of 50 ul. The program was the same as the first amplification without the initial extension and for the calculated number of cycles. Finally, re-amplified DNA was cleaned up using 1.8x volumes AMPureXP beads and eluted in 30 ul. Samples were quantified in the QuBit, size profiles examined in the Bioanalyzer using the DNA High Sensitivity DNA Kit (Agilent, Part No. 5067-4626). Samples were pooled and sequenced in a NovaSeq by Novogene (Cambridge, UK).

### Bulk ATAC-seq

#### Tn5 production

Methods were adapted from Hennig et al.^87^ as follows: The pETM11-Sumo3-Tn5_E54K,L372P_ plasmid was obtained from EMBL-Heidelberg and transformed into *Escherichia coli* BL21 (DE3) Gold cells (Agilent). Bacterial cultures were grown at 30°C to a density of OD_600_=0.6-0.8 and protein expression was induced with 0.5 mM isopropyl-β-D-thiogalactoside (IPTG). Prior to induction, cultures were cooled and maintained at 20°C for protein expression overnight. Cell pellets were harvested by centrifugation for 10 m at 4000 rpm and then resuspended in lysis buffer (50 mM Tris-HCl pH 8.0, 20mM Imidazole, 0.5 M NaCl, 10% Glycerol, 1mM TCEP, 1U/ml Benzonase, 1 tablet/50ml Protease inhibitor tablets (cOmplete, Roche) and lysed with sonication. The cell lysate was then centrifuged for 30 m at 80,000 x *g*. The supernatant was collected and applied onto a 5ml Ni-Sepharose column (Cytiva). The column was washed with 10 Column Volumes wash buffer A (50mM Tris-HCl pH 8.0, 20 mM Imidazole, 0.5 M NaCl,10% Glycerol, 1mM TCEP). Bound proteins were eluted with buffer A containing 300mM Imidazole. Fractions containing His-Sumo3-Tn5_E54K,L372P_ were pooled and incubated overnight at 4°C with SenP2 protease to remove the fusion tag. Next day, the sample was diluted six times and applied onto a 5ml Heparin HP column pre-equilibrated in buffer B (50mM Tris-HCl pH 8.0, 0.05 M NaCI, 1mM TCEP). Bound Tn5 protein was eluted with a linear gradient to 100% buffer B containing 1M NaCl, concentrated and loaded onto a Superdex 200 Increase 10/300 GL column (Cytiva) equilibrated in buffer C (50 mM Tris pH 8.0, 150 mM NaCl, 1 mM TCEP and 5% glycerol). Fractions corresponding to the Tn5 peak were pooled, concentrated to 10μM and flash frozen in liquid nitrogen.

#### Tn5 assembly

Dilution and oligo assembly of Tn5 was performed as previously described.^28,88^ In brief: purified protein was diluted to 4 μM in Dilution Buffer (50 mM Tris, 100 mM NaCl, 0.1 mM EDTA, 1 mM DTT, 0.1% NP-40, and 50% glycerol). Two independent oligo mixes were assembled (Mix A, Mix B), each with 5 μl of 100 μM Tn5MEREV oligo, 5 μl of 100 μM of either Tn5_1 (Mix A) orTn5_2_ME_comp (Mix B), and 40ml of nuclease-free water by incubating each mix in a PCR thermocycler as follows: 95°C for 3 m, 65°C for 3 m and ramp down to 24°C at a rate of -1C/min. Once annealed, Mix A and Mix B were combined with 100 μl glycerol to create a 5 μM, 50% glycerol adaptor mix. Equal parts of diluted Tn5 transposase and adaptor mix were mixed and incubated at 25°C for 60 min. For oligo sequence see Table S1

#### Transposition and library generation

Wells were washed and cells were dissociated using 0.5 ml Accutase per well of a 6 well plate incubated 5 min at 37°C. Cells were washed in PBS, and 50,000 cells per condition and replicates were used for ATAC-seq following established protocols^28,89^ (for sequences used see Table S1) and as described for CaTS-ATAC. After transposition, DNA was directly purified and used for library preparation (as described for CaTS-ATAC).

### Crosslinked and TF-Sorted RNA-seq

Samples for CaTS-RNAseq were collected in the same way as for CaTS-ATAC but all buffers were supplemented with 1 μL/ml of RNasin Plus (Promega, Cat. No. N2615). After fixation and quenching, samples were stained for intracellular TFs, also as described in “Flow cytometry of intracellular markers” with RNasin-supplemented buffers.

Cells were sorted into 0.5 ml PBS with 0.5% BSA and 1 μl/ml of RNasin Plus. RNA extraction from fixed samples were performed with the RecoverAll RNA/DNA Isolation Kit (Thermo Fisher Scientific, Cat. No. AM1975) with the modifications described in Amamoto et al.^90^ In brief, sorted cells were spun down at 3000 g for 7 min at 4°C and as much supernatant as possible was removed (leaving 40-50 μl). 100 μl of Digestion Buffer previously mixed with 4 μl of Protease mix was added to each sample and resuspended. The tubes were incubated at 50°C for 3 h and then stored at -80°C. RNA isolation was performed as per manufacturer’s instructions, eluting in 17 μl of Nuclease-free water.

Due to low quality and limiting amounts, the maximum amount (10.5 μl) was used for library preparation using the SMART-Seq HT kit (Takara, Cat. No. 634437) followed by Nextera XT DNA Library Preparation Kit (Illumina, Cat. No. FC-131-1096).

### ATAC-seq processing

Data was processed using the nf-core atacseq pipeline (https://nf-co.re/atacseq) with the following options: –genome mm10–macs_fdr 0.00001 –skip_diff_analysis –min_reps_consensus 2 -r 1.2.0. Peaks were further filtered out if they didn’t pass qval < 0.00001 in at least 1 sample (qval generated from MACS peak calling in each sample).

### ATAC-seq differential expression analysis

Read counts within the consensus intervals generated by featureCounts were used as input for DESeq2.^83^ Principal Component Analysis was performed using the top 30000 most variable elements and coloured by different sample metadata.

To assess the cell type specific accessibility for the same NP cell types at different SAG concentrations, all by all differential expression was performed for all conditions at day 5. Number of differentially accessible intervals plotted (fold change > 2, basemean > 100) as a bar graph, and individual elements are plotted color-coded by their p-adjusted values for specific pairwise comparisons.

Selection of differentially accessible elements across cell types and timepoint was performed by pairwise DESeq2 analysis between any two cell types within a timepoint and any two timepoints for the same cell type. Elements that fulfilled padj < 0.01 & abs(log2FoldChange) > 2 & baseMean > 100 were selected for subsequent clustering.

### ATAC element clusters by kmeans

Varianced stabilized transformed data generated using DESeq2 were used as input to identify clusters of elements with the same dynamics. Clustering was performed using kmeans with a high number of centers, 30, and subsequently re-grouping clusters of very similar dynamics using hclust and target of 9 final clusters. This was chosen as independent iterations resulted in reproducible clusters and dynamics.

### Analysis of published ChIP-seq data

Processing of published ChIP-seq datasets was for NKX6.1, NKX2.2, OLIG2,^16^SOX2 and FOXA2^13^from neural embryoids with SAG were processing using the nf-core chipseq pipeline as follows: nextflow run nf-core/chipseq –input design.csv –single_end –genome mm10 -profile crick -r 1.1.0.

FOXA2 from endoderm differentiations^50^ were process with the nf-core atacseq pipeline due to lack of input samples. nextflow run nf-core/atacseq –input design.csv –single_end –mito_name false –genome mm10 -profile crick -r 1.1.0.

### Footprinting analysis

BAM files for merged replicates were used as input for TOBIAS ATACorrect,^33^ followed by TOBIAS Footprint. TOBIAS BINDetect was run on all samples combined using the following the JASPAR2018, HOCOMOCO and Taipale databases.^34–36^

Motifs that were amongst the top 5% highest absolute fold change or 5% smallest pvalues in each pair-wise comparison were selected. Motifs were grouped in archetypes.^37^ The most variable archetypes for the desired comparison (either p0-1, p2, pM at day 5 and 6, or all conditions) were selected and RNA expression for each TF that is associated with the archetype was compared to the motif score for that archetype. This correlation analysis between motif score and RNA expression was used to find candidate TFs driving the chromatin changes observed.

### RNA-seq data processing

Data was processed using the nf-core rnaseq pipeline with the following options: –star_index ’star_ercc_mm10_genome’ –gtf’mm10.refGene_wERCC.gtf’ –fc_group_features_type gene_id –fc_extra_attributes gene_id -r 1.4.2. Gene counts were obtained from featureCounts with the option ignoreDup=TRUE to try and remove PCR duplicates. Samples were excluded from downstream analysis based on a number of QC criteria particularly applicable to this type of low input samples from PFA-fixed cells including: too small proportion of the library being of mouse origin (excess spike-in representation), large number of overrepresented sequences, and loss of dynamic range in spike-in quantification.

## Quantification And Statistical Analysis

Details for bioinformatic data analysis and software used are detailed in each method section. No additional statistical analyses were conducted. All code has been deposited at https://github.com/MJDelas/Neural_DV_ATAC, including parameters and versions for all software used. Figure legends contain number of replicates and details of data plotted (mean, s.e.m).

## Supplementary Material

Supplementary figures

Table S1

## Data Availability

Sequencing data (CaTS-ATAC, live Omni-ATAC, RNA-seq and bulk ATAC-seq) have been deposited at GEO and are publicly available as of the date of publication. Accession numbers are listed in the key resources table. Accession number for the SuperSeries is GSE204921. Microscopy data reported in this paper will be shared by the lead contact upon request. All original code has been deposited at https://github.com/MJDelas/Neural_DV_ATAC and is publicly available as of the date of publication. Custom code for lineage tracing quantification are available at https://github.com/MJDelas/tdTom_LinTracing. Any additional information required to reanalyse the data reported in this paper is available from the lead contact upon request.

## In brief

Delás et al. provide evidence that spinal cord neural progenitors use two different chromatin regulatory strategies to control differential gene expression in response to the morphogen Shh. This highlights a conserved role of the pioneer factor FOXA2 between endoderm and neural cells, suggesting potential evolutionary relationships between these cell types.

## Highlight

- CaTS-ATAC reveals chromatin accessibility from intracellular marker cell sorting
- Neural progenitors employ two chromatin regulatory strategies in response to Shh
- Most progenitors share accessibility across the same regulatory elements
- FOXA2 mediates a “differential accessibility” strategy in the most ventral progenitors
