# Supplementary material for: Developmental cell fate choice in neural tube progenitors employs two distinct cis-regulatory strategies
Source: Dev Cell. Author manuscript; Available in PMC 2023 Mar 9. (PMC7614300; doi:10.1016/j.devcel.2022.11.016)
Supplement: Supplementary figures [file EMS170713-supplement-Figure_S1.pdf]

Developmental Cell, Volume *58*

Supplemental information

Developmental cell fate choice in neural tube progenitors employs two distinct *cis*-regulatory strategies

M. Joaquina Delás, Christos M. Kalaitzis, Tamara Fawzi, Madeleine Demuth, Isabel Zhang, Hannah T. Stuart, Elena Costantini, Kenzo Ivanovitch, Elly M. Tanaka, and James Briscoe

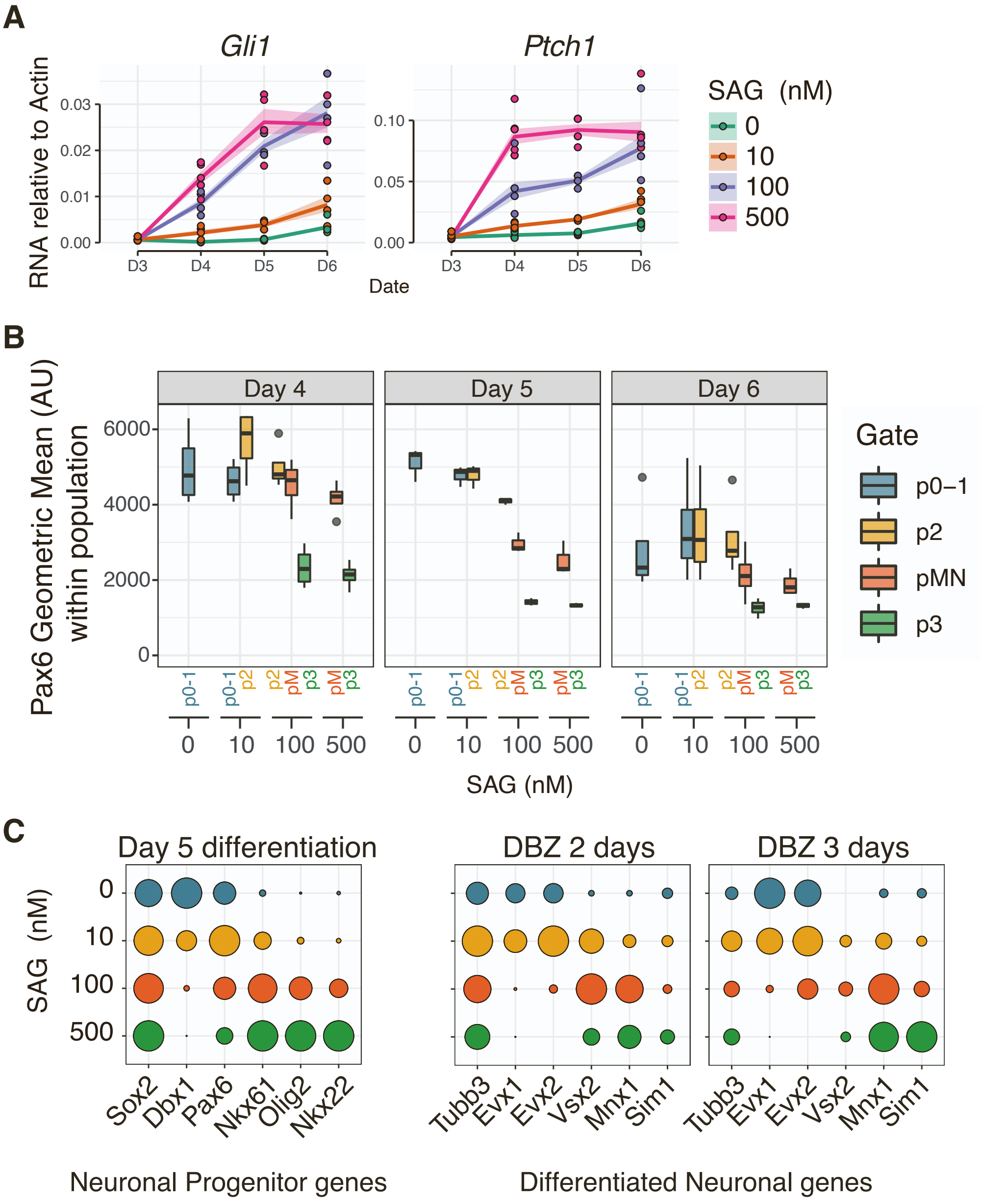

Figure S 1: A cellular model of ventral spinal cord neural progenitors recapitulates aspects of in vivo patterning, Related to Figure 1

(A) Relative expression (RT-qPCR) for Shh responsive genes show a SAG concentration-dependent response. N=4 biological replicates (independent differentiations). Line represents average; shaded area, s.e.m.

(B) Pax6 levels recapitulate the lower expression in pMN compared to p0-1 and p2 observed for this protein in vivo. Pax6 is not expressed in p3 NPs. N=4 biological replicates (independent differentiations).

(C) Relative expression (RT-qPCR) for progenitors and neuronal markers at Day 5 (predominantly progenitors) and 2 or 3 days after neuronal induction with Notch inhibitor dibenzazepine (DBZ) show the expected enrichment for dorsoventral progenitors and neuronal markers as a function of SAG concentration. N=2 biological replicates (independent differentiation). Area of the dots represents the average relative gene expression.

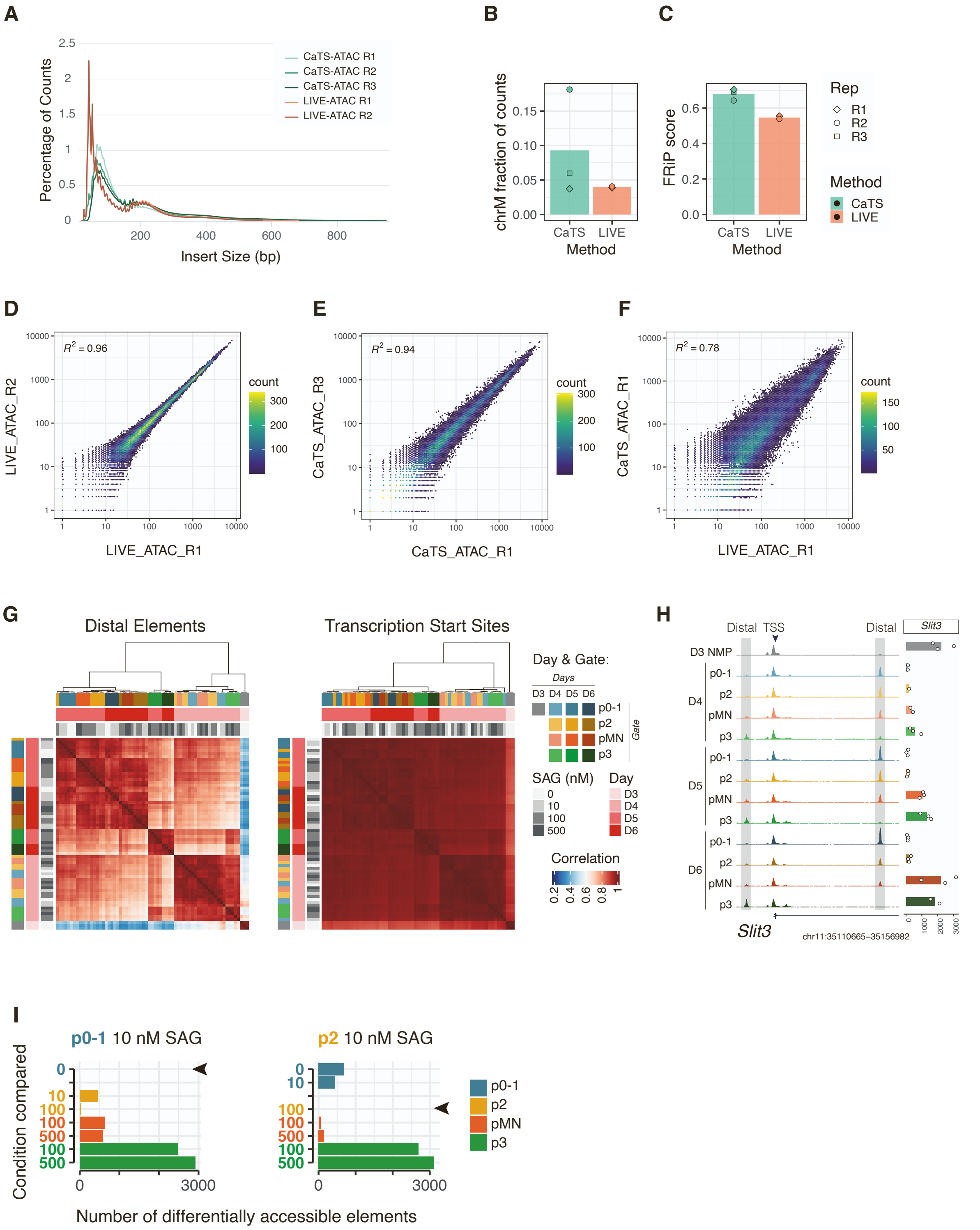

Figure S 2: Cell type specific ATAC-seq characterization, Related to Figure 2.
(A) Insert size profiles for CaTS-ATAC pMN samples compared to live Olig2 reporter-sorted cells assayed by standard Omni-ATAC-seq shows a small depletion of smallest size fragments, consistent with other reports.
(B) The fraction of mitochondrial DNA captured by CaTS-ATAC is on average similar to Omni-ATAC, and (C) so is the Fragments of Reads in Peaks (FRiP). N=3 CaTS-ATAC, N=2 Omni-ATAC.
Correlation of normalized counts across all consensus elements for two replicates of (D) Omni-ATAC from live cells and (E) replicates of CaTS-ATAC shows extremely high correlation.
(F) Correlation between methods is high although lower than within methods.
(G) Sample to sample correlation based on distal elements shows sample clustering by cell type. Correlation based on TSS is less cell type specific.

(H) Example locus showing broad accessibility across the promoter in all samples and cell type specific variations in accessibility at distal elements.
(I) Number of differentially accessible elements between the indicated samples and all other conditions reveals few or no differentially accessible elements between the same cell type from a different concentration of SAG.

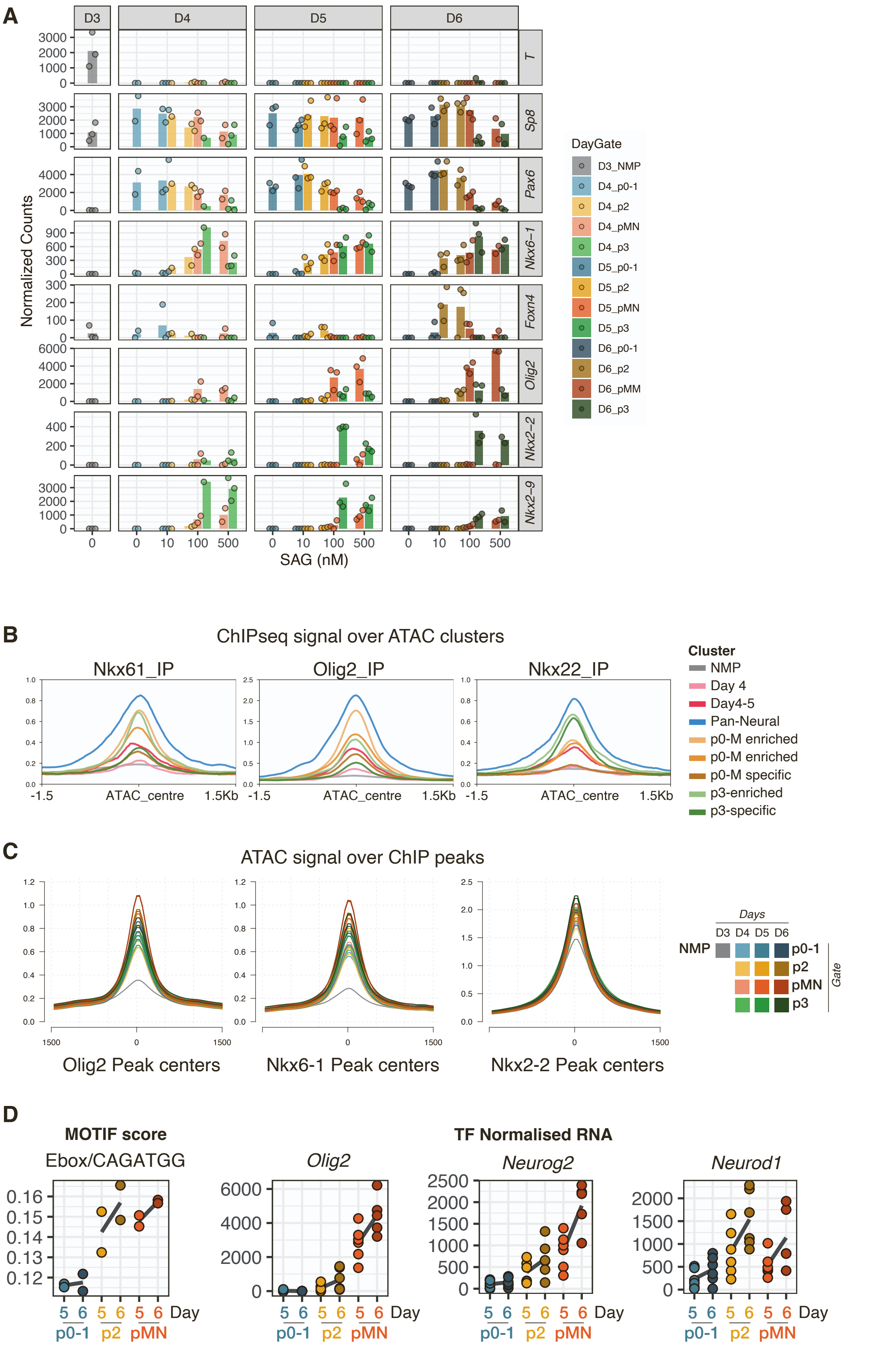

Figure S 3: **A shared accessibility landscape for p01-, p2 and pM, Related to Figure 3.**
(A) RNA-seq normalized gene counts for key marker genes validates cell type identity of sorted populations.

(B) Normalized ChIP-seq coverage of each ChIP-seq data set over the groups of elements belonging to each ATAC clusters from Fig 3B shows binding of all TFs to pan-neural and cell type specific clusters.
(C) Normalized accessibility coverage of each sample over the peak called regions for each ChIPseq data set shows accessibility of all NPs over the regions bound by all TFs.
(D) Footprinting score for the archetype Ebox/CAGATGG is higher in p2 and pM. Normalized RNAseq expression for the top correlated three genes associated with this motif reveals their potential contribution to the footprint signal in different cell types.

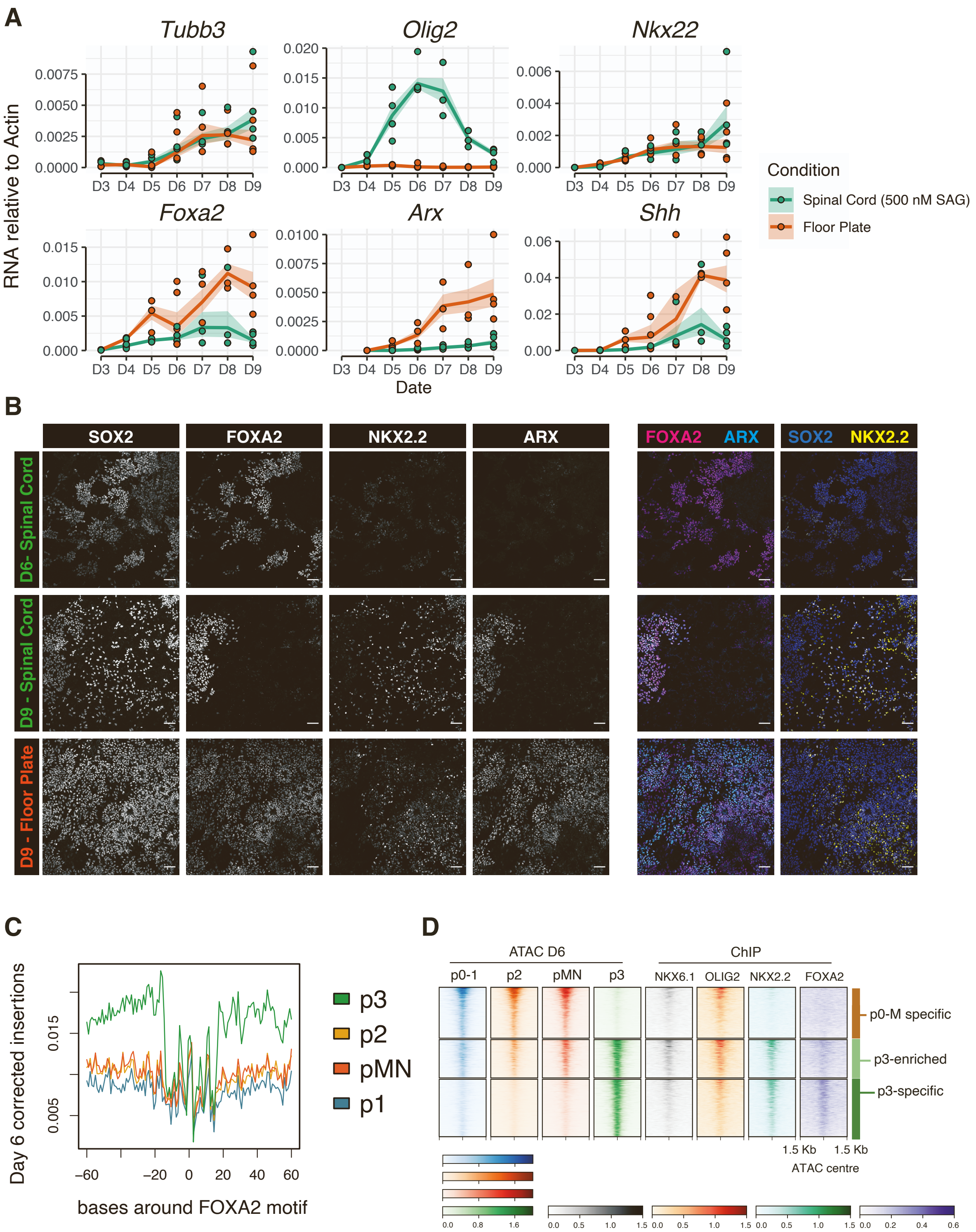

Figure S 4: Spinal cord neural differentiation generates minimal amounts of floor plate and Foxa2 binding p3 NPs, Related to Figure 4.

(A) Relative mRNA (RT-qPCR) for the genes indicates shows clear induction of floor plate (FP) markers in FP conditions and minimal induction in spinal cord neural differentiations. N=4 biological replicates.

(B) Immunohistochemistry staining for shows robust induction of FP markers (ARX) in FP conditions at Day 9, whereas Spinal Cord conditions lead to only small groups of cells expression ARX at Day 9. No ARX was detected at Day 6 in Spinal Cord conditions. Scale bar, 50 μm.

(C) Representative metaplot of corrected insertions over the FOXA2 motif across all accessible regions for all NPs at Day 6 shows a footprint in p3 NPs.

(D) Coverage heatmap for ATAC and ChIP-seq for the p0-M specific, the p3-enriched and the p3-specific cluster shows binding of NKX2.2 and FOXA2 to p3-specific sites.

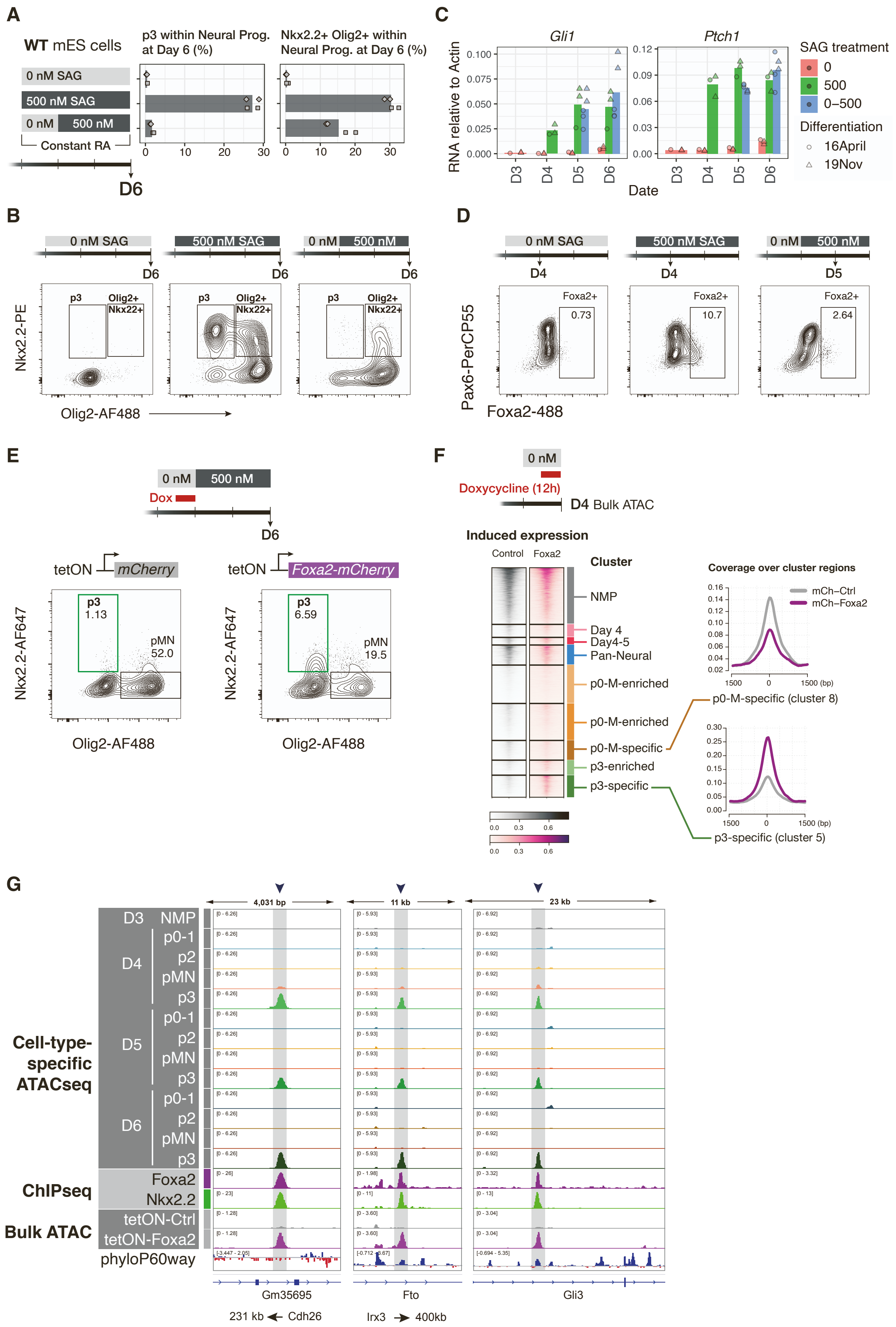

Figure S 5: Delayed SAG abolishes p3 generation due to lack of Foxa2 induction, Related to Figure 5.

(A) Generation of p3 at day 6 is greatly impaired if SAG administration is delayed by 24h (left graph). Differentiations do express Nkx2.2 but co-expressed with Olig2 (right). N=2 biological replicates (independent differentiations) and 2 independent samples from each differentiation.
(B) Representative flow cytometry plots for the data quantified in (A). Gated on SOX2+ live neural progenitors.
(C) Relative expression of Shh target genes shows cells respond to signalling in the delayed SAG regime (“0-500”). Same N as (A).
(D) Flow cytometry of Foxa2 intracellular antibody staining without SAG administration of 24h after SAG administration shows reduced Foxa2 induction in the SAG delayed regime.
(E) Doxycycline-induced expression of Foxa2 for 12h in the SAG delayed regime rescues p3 generation at day 6.
(F) Bulk ATAC seq coverage after overexpression of Foxa2 or control for the ATAC clusters identified in Fig 3B (left). Metaplot of normalized coverage comparing ATAC in Foxa2 and control overexpressed for cluster p0-M-specific and p3-specific show increased accessibility of Foxa2 overexpression in p3-specific sites (right).
(G) Example loci of the heatmap shown in Fig 5D, depicting regions specifically open in p3 cells, accessible in response to induced Foxa2 (but not control) and bound by FOXA2 itself and NKX2.2.

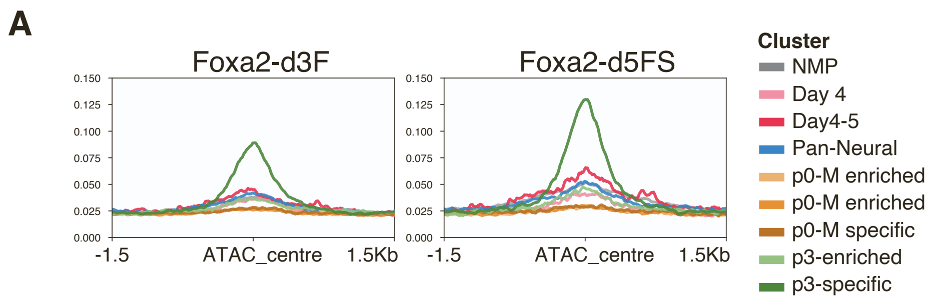

Figure S 6: Binding of endodermal Foxa2 to ventral neural sites, Related to Figure 6.
(A) Normalized Foxa2 ChIP-seq coverage over the ATAC-seq clusters identified in Fig 3B shows accessibility in p3-specific sites.
